# Integrative Biomimetics of Autonomous Hexapedal Locomotion

**DOI:** 10.3389/fnbot.2019.00088

**Published:** 2019-10-23

**Authors:** Volker Dürr, Paolo P. Arena, Holk Cruse, Chris J. Dallmann, Alin Drimus, Thierry Hoinville, Tammo Krause, Stefan Mátéfi-Tempfli, Jan Paskarbeit, Luca Patanè, Mattias Schäffersmann, Malte Schilling, Josef Schmitz, Roland Strauss, Leslie Theunissen, Alessandra Vitanza, Axel Schneider

**Affiliations:** ^1^Department of Biological Cybernetics, Faculty of Biology, Bielefeld University, Bielefeld, Germany; ^2^Cognitive Interaction Technology: Center of Excellence, Bielefeld University, Bielefeld, Germany; ^3^DIEEI: Dipartimento di Ingegneria Elettrica Elettronica e Informatica, Università degli Studi di Catania, Catania, Italy; ^4^Mads Clausen Institute, University of Southern Denmark, Sønderborg, Denmark; ^5^Institut für Entwicklungsbiologie und Neurobiologie, Johannes Gutenberg-Universität, Mainz, Germany; ^6^Institute of System Dynamics and Mechatronics, Bielefeld University of Applied Sciences, Bielefeld, Germany

**Keywords:** motor control, walking, compliance, leg coordination, proprioception, load sensing, internal model, motor learning

## Abstract

Despite substantial advances in many different fields of neurorobotics in general, and biomimetic robots in particular, a key challenge is the integration of concepts: to collate and combine research on disparate and conceptually disjunct research areas in the neurosciences and engineering sciences. We claim that the development of suitable robotic integration platforms is of particular relevance to make such integration of concepts work in practice. Here, we provide an example for a hexapod robotic integration platform for autonomous locomotion. In a sequence of six focus sections dealing with aspects of intelligent, embodied motor control in insects and multipedal robots—ranging from compliant actuation, distributed proprioception and control of multiple legs, the formation of internal representations to the use of an internal body model—we introduce the walking robot HECTOR as a research platform for integrative biomimetics of hexapedal locomotion. Owing to its 18 highly sensorized, compliant actuators, light-weight exoskeleton, distributed and expandable hardware architecture, and an appropriate dynamic simulation framework, HECTOR offers many opportunities to integrate research effort across biomimetics research on actuation, sensory-motor feedback, inter-leg coordination, and cognitive abilities such as motion planning and learning of its own body size.

## Introduction

In neurorobotics, animals are more than just a source of inspiration. They also serve as reference systems for many, apparently disparate computational competences such as: (i) reliable, resource-efficient, parallel and/or de-centralized computing in real time; (ii) autonomous, fast and robust decision-making in complex environments; and (iii) flexible coordination and control of many degrees of freedom (e.g., Ijspeert, 2014). To date, research has tended to all of these computational competences of animals, and neurorobotics has seen many successful abstractions and implementations of selected neural mechanisms.

Natural locomotion behavior of multi-legged animals is an example of intelligent interactive behavior where all of the mentioned competences are equally relevant. With regard to bio-inspired walking robots with six or more legs, early research concentrated on mechanical design (e.g., Pfeiffer et al., 1995) and force control (e.g., Devjanin et al., 1983; Schneider et al., 1995). This line of research has been developed further continuously, including bio-inspired approaches to system design such as evolutionary optimization (e.g., Bartsch et al., 2012). Concerning control, the implemented biomimetic approaches may be assigned to one of two major streams.

The first of these streams emphasized the principle of modular sensorimotor control with a focus on sensory feedback (e.g., Pfeiffer et al., 1995; Espenschied et al., 1996; Schneider et al., 2006). In many cases, the sensorimotor control modules were implemented by use of artificial neural networks (e.g., Berns et al., 1994; Schmitz et al., 2008; von Twickel et al., 2012), thus requiring learning prior to operation (e.g., Ilg and Berns, 1995) and/or during operation (e.g., Manoonpong et al., 2008). For example, the performance on difficult terrain can be improved through machine learning techniques (e.g., Bartsch et al., 2012; Goldschmidt et al., 2014).

The second stream of biomimetic approaches emphasized experimental findings on biomechanics and neural oscillators and implemented different forms of rhythmic pattern generators for hopping (e.g., Altendorfer et al., 2001) or walking (e.g., Arena et al., 2012), including highly modular approaches based on mechanical coupling alone (Owaki et al., 2017). A more theoretical approach within this stream of research also succeeded in exploiting chaotic properties of neural oscillatory networks (Steingrube et al., 2010). Both streams of research have at least partially included results derived from behavioral experiments, either by implementing particular motion patterns (e.g., Klaassen et al., 2002) or a continuum of free gaits based on the rules governing inter-leg coordination (e.g., Espenschied et al., 1996; Schmitz et al., 2008), but also theoretically derived criteria (e.g., Fielding and Dunlop, 2004). This plethora of approaches has been reviewed with respect to the mutual benefits of biology and engineering in general (e.g., Ritzmann et al., 2000; Ayers et al., 2002), and adaptive control strategies for multi-legged robots in particular (e.g., Arena and Patanè, 2009; Aoi et al., 2017).

**Figure 1:**
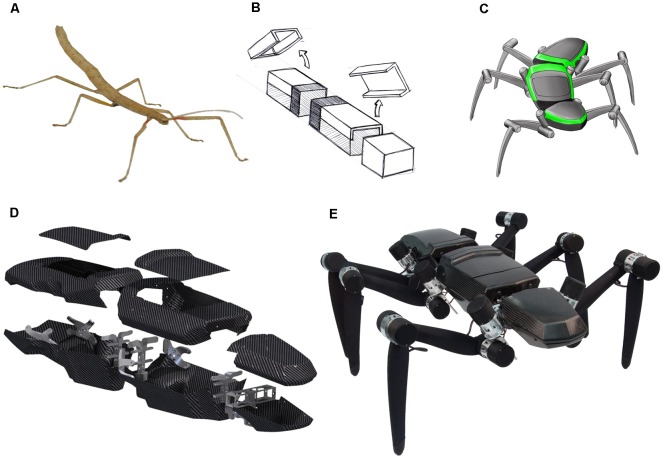
HECTOR—from bio-inspiration to a physical robot. **(A)** The Indian stick insect *Carausius morosus* served as a template for the robot design. Especially, the relative distances of the leg onsets, the alignment of leg joint axes and the subdivision into three body segments were transferred during the design process. **(B)** Early abstraction of the three body segments prothorax, mesothorax and metathorax as compartments for the accommodation of “head-related” sensors, embedded computer system and battery, respectively. **(C)** First design sketch (Folkwang University of the Arts, Essen, Germany) of the hexapod robot considering the general shape demands from panels **(A,B)**. **(D)** 3D-CAD-rendering of the light-weight, self-supporting body segments with an exoskeleton made of carbon fiber composites (manufactured at the Leibniz-Institute of Polymer Research in Dresden, Germany). Only few metal parts are included for directing the leg forces into the housings. **(E)** Photo of HECTOR. The body length of the assembled robot is 95 cm. The total mass is 13 kg. Approximately 7 kg of the mass comes from the 18 compliant joint drives in the legs.

In spite of the remarkable achievements of individual research efforts, the integration of multiple, equally well-developed competences in a single robotic platform is still a challenge. Here, we argue that a key challenge of neurorobotics is the necessity to integrate concepts from different fields of engineering and neuroscience—and the ensuing necessity to have appropriate robotic integration platforms. To illustrate how we envisage such collaborative, multi-competence effort on a single robotic integration platform, we use the hexapedal walking robot HECTOR (Figure 1; Schneider et al., 2014; Paskarbeit et al., 2015). As a research platform, HECTOR is special because it offers many opportunities for integrating concepts of neuroscience and engineering alike. It has 18 highly sensorised, compliant actuators, a light-weight exoskeleton (Figures 1D,E), and a hardware architecture that is suitable for de-centralized control. Together with a summary of our current understanding of motor flexibility in HECTOR’s biological paragon, the stick insect (Figure 1A), we provide examples of various aspects of natural motor control. In doing so, we close the loop between multiple embodied sensory systems and compliant actuators by different sensorimotor mechanisms of inter-leg coordination, including cognitive abilities such as motion planning.

The results presented in this article are grouped into six sections, with each section focusing on a different aspect of intelligent adaptive walking systems in biology and technology. Together, these sections provide an integrative view of a biomimetic walking system, ranging from: (I) compliant actuation; (II) distributed proprioception of posture and load; to (III) the particular role of body-substrate interaction; (IV) spatial coordination of multiple legs. Based on these aspects of de-centralized control, we (V) discuss different modular control concepts for adaptive coordination of multiple legs, including the role of internal models in context-dependent coordination of a complex body. Finally, we expand the cognitive repertoire of HECTOR by (VI) a neural network model that can form an internal body-representation for decision-making on the grounds of learned own motor abilities. Each one of the six facets will be introduced by a current view on biological systems and emphasize the behavioral relevance for an animal. This will be complemented by a specific suggestion on how to abstract biological insights and implement at least some of them in a technological framework. Last but not least, each section will point out why the contribution is relevant for an integrative hardware model of multi-legged locomotion and, thus, a holistic view on flexibility and robustness of multi-legged walking in animals and machines.

## Muscles and Compliant Actuation

All biological locomotion systems are compliant, simply for the fact that biological actuators (i.e., muscles and tendons) are made of deformable macromolecular structures that may drive deformation of tissues or move adjacent limb segments connected by articulated joints. A common view is that muscle-tendon systems in animals are tuned to serve a particular purpose, either in accelerating or decelerating a body part or by transmitting forces efficiently (Dickinson et al., 2000; Alexander, 2003). As such, compliance in biological motion may store and release energy in a passive manner but may also contribute actively to improve movement efficiency. While both passive and active compliance is relevant for resource efficiency, a further benefit of passive compliance is safety in the sense that it allows dissipation of energy, for example during the impact of a foot at touch-down.

### Compliance of Muscle

A muscle can be thought of as a force generator that is controlled by the central nervous system (CNS). The forces actively generated by the respective muscles, as well as the resulting torques at the actuated joints, are non-linear functions of the activation and contraction dynamics of muscles (Zajac, 1989; Zakotnik et al., 2006), as well as of the mechanical integration of the musculotendinous complex into the joint. The activation dynamics represents the time course of the chemical activation processes within muscle fibers (e.g., calcium dynamics). In case of vertebrate muscle, where muscle activation is largely dependent on the number of motoneurons recruited, muscle activation dynamics is typically described by a first-order non-linear differential equation (Zajac, 1989; Buchanan et al., 2004). In insects, where muscles are often innervated by very few motoneurons and single twitches can last very long, higher-order nonlinearities are used (Zakotnik et al., 2006; Wilson et al., 2013; Harischandra et al., 2019). The contraction dynamics represents the influence of muscle length and shortening velocity on the active force generation of a muscle (Hill, 1938; Aubert, 1956; Zajac, 1989; Romero and Alonso, 2016). Activation dynamics and contraction dynamics are assumed to be independent of each other although this has been discussed controversially (Rack and Westbury, 1969). Muscles are connected to segments *via* soft tissue e.g., tendons which also show a non-linear compliant behavior for which different formulations have been proposed (e.g., Hatze, 1974; van Soest and Bobbert, 1993; Thelen, 2003). The mechanical effect of muscle forces onto a joint is further influenced by the dependence of the lever arm length on joint angle, and non-linear damping due to the soft tissue in which the actuator is embedded.

Since muscles can generate active forces in one direction only, joint actuation has to be accomplished at least by an antagonistic pair of muscles in which one of the players can also be replaced by a passive elastic structure. Due to the presence of at least two muscles per joint and due to additional degrees of freedom arising from nervous activation of muscles, the mechanical function of any muscle-tendon system may vary greatly depending on the timing and magnitude of its recruitment (e.g., Sawicki et al., 2015). The level of co-activation of antagonistic muscles allows the regulation of joint stiffness (Hogan, 1984; Gribble et al., 2003; Zakotnik et al., 2006). The mixture of co- and reciprocal activation, for instance, allows an almost separate adjustment of compliance and joint angle, at least in certain intervals of the angular working range (Annunziata et al., 2011; Annunziata and Schneider, 2012).

Therefore, with regard to a particular motor task, the CNS is responsible for controlling not only the movement itself, but also the compliance of the system, particularly for maintaining stability during interaction tasks that involve impacts or other interaction forces between the body and an external object (e.g., as in manipulation tasks).

### Compliance in Biomimetic Actuators

In the technical domain, a variety of damped and undamped compliant actuation systems have been developed that can be subsumed under the term “variable impedance actuators.” Recently, Vanderborght et al. (2013) have categorized this family of actuators into “*active impedance by control*,” “*inherent compliance*” (passive compliance), “*inherent damping*” and “*inertial*” actuators. Of these, *inherent compliance* and *inherent damping* actuators have the advantage of not requiring any active control of compliance (e.g., by a second actuator for adjusting joint stiffness), at the cost of having a mechanically fixed impedance behavior.

Inherent damping means that a passive visco-elastic element reduces oscillations of the compliant system when mechanically excited, for example in response to a collision with an obstacle. In contrast to *active impedance by control* actuators, passive actuator systems have no bandwidth-limitation of the elastic effect. In order to exploit the advantages of adjustable impedance, passive actuators may be operated in a “hybrid fashion,” where compliance can be adjusted by control, as opposed to modification of the mechanical properties. In this way, mechanically passive actuator systems may be used to implement muscle-like actuation (Annunziata and Schneider, 2012), even though muscles are not passive systems. The combination of inherent compliance and inherent damping leads to a well manageable behavior of HECTOR’s joint drives. However, it must be stressed that the control of compliant structures with high dynamic bandwidth, in general, is challenging. Solution approaches contain passivity based impedance control (Albu-Schäffer et al., 2007), classical impedance control (Hogan, 1985) or hybrid impedance control (Anderson and Spong, 1988).

### The Compliant Joint Drives of HECTOR

The ability of physical interaction with the environment is a key feature of animal locomotion, involving repeated impacts of the feet on the ground, mechanical coupling of a variable number of legs through body and substrate and, as a consequence, discontinuous changes of force- and torque interactions among the individual joint actuators. The manifold of mechanosensory information arising through these bodily interactions is a foundation for sensory, (event)-driven walking controllers such as Walknet (as originally described by Cruse et al., 1995, see below). For the control of mechanical interaction of body and substrate, a reliable estimate of joint torques during resisted actuation is desirable. Much like the force estimate of isometric muscle contractions requires the combination of Golgi tendon organs and the compliant tendon, here, the combination of a sensor and the serial elasticity of the compliant actuator is needed.

**Figure 2:**
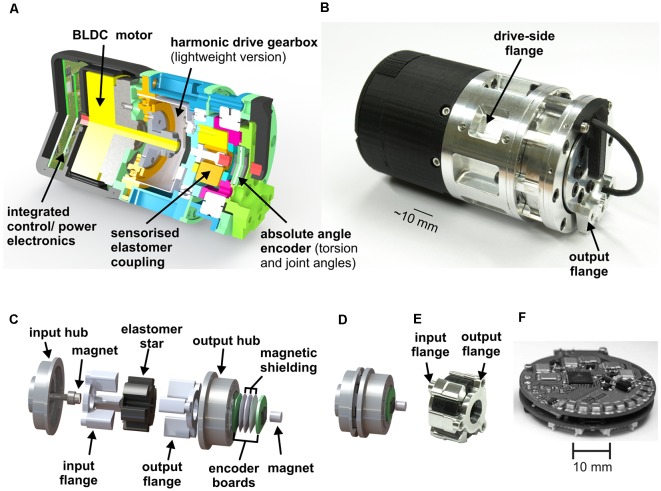
Compliant joint drive with elastomer coupling. **(A)** The section view of an elastic joint drive of HECTOR. It contains a power- and control-electronics stack, a brushless DC motor, a light-weight harmonic drive gearbox, and a sensorised elastomer coupling with two position encoders. **(B)** Photographic depiction of the elastic joint drive with mounting points for adjacent segments. Stable force transmission is achieved by the positive locking of segment and seating (input flange and output flange). **(C)** Explosion view of the coupling shown in panel **(D)**. The input flange is linked to the output of the gearbox. It connects to the input hub that carries three teeth, each of which extends into a corresponding notch of the elastomer. The remaining three notches in the elastomer are held by the three teeth of the output hub which, in turn, is fixed to the output flange. The elastic torsion of the elastomer and the resulting twist between input and output hubs is measured by a Hall-effect position sensor (after Paskarbeit et al., 2013, with permission). **(D)** View of the elastomer coupling, as integrated into the drive. **(E)** Photo of input and output flange, together with elastomer star. **(F)** Image of the power- and control-electronics stack which is mounted in the back of the drive (after Paskarbeit et al., 2013, with permission).

The compliant actuators of HECTOR belong to the *inherent compliance* category: they use an elastomer coupling as the compliant element. Because the elastomer has visco-elastic properties, it introduces an *inherent damping* component into the actuator, too, other than a set of steel springs would do. Figure 2A shows a sectional view of the fully integrated, compact and compliant drive system which is used in each one of the 18 leg joints of HECTOR. Figure 2B shows a photo of the drive. The weight of each drive is below 0.4 kg. As a result, about 55% (7.2 kg) of the robot’s total weight (13.0 kg) is constituted by its joint actuators. The drive includes small-scale electronics, integrated as a PCB stack (Figure 2F). The PCB stack contains power-, communication- and control-electronics. It is software-controlled by an integrated 8-bit microcontroller. The core of the actuator is a brush-less DC motor, driving a light-weight harmonic drive gearbox. The short installation length of motor and gearbox allows for the small dimensions of the entire system (length ~90 mm, diameter ~50 mm). Motor and gearbox are followed by an elastomer coupling (Figures 2C–E), making it a serial elastic actuator (Pratt and Williamson, 1995). The main reason for favoring an elastomer coupling over a steel spring coupling was the fact that it can be scaled down to a diameter of 20 mm, allowing for compact integration (Figure 2D). The input flange with its hub is attached to the output of the gearbox. The output flange of the coupling with its hub is mechanically connected to the output of the joint. The torsion between input and output hub is mediated by two sets of three teeth (photo in Figure 2E), gearing into the six lobes of the elastomer star. The elastomer star was used as an inlay, i.e., not bonded to the metal teeth of the hubs. In principle, it can be bonded to the teeth as well. For torsion measurements at the elastomer coupling, it is equipped with a magnet and Hall sensor ensemble. A second one of these ensembles measures the output angle of the drive. A characterization of the non-linear behavior of the compliant element as well as a suitable fit function for a system model can be found in Paskarbeit et al. (2013).

## Distributed Proprioception of Posture and Load

All animals physically interact with their environment, as any overt behavior requires the generation of force: force to accelerate the own body’s center of mass (locomotion), force to deform or displace external structures (manipulation), and force to accelerate a limb in order to generate or acquire information through limb movement (signaling and active sensing). As a consequence, the control of force is a fundamental requirement of purposeful, interactive behavior. The sensory modality involved is proprioception, the mechanoreception of force and posture (for review, see Tuthill and Azim, 2018). Two hallmarks of proprioception are: (i) the intimate relationship between the process of sensory transduction and the biomechanics of the surrounding body tissue; and (ii) its distributed nature, i.e., the fact that each and every body part is equipped with different mechanoreceptors. The combination of these two aspects implies that the entire body of an animal essentially serves as one complex proprioceptive organ. In the following section, we will review some general aspects of distributed proprioception in insects, with a focus on load sensing in locomotion. In conjunction with these considerations, we will explain the concept of distributed proprioception in HECTOR.

### Distributed Proprioception in Insects

**Figure 3:**
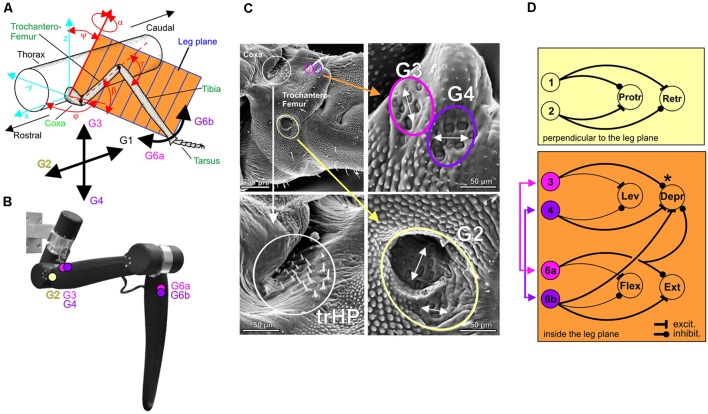
Distributed proprioception in the insect walking system. **(A)** Schematic of the reference frame for force detection by campaniform sensilla (CS) of an insect leg. For a given axis orientation (φ, ψ) and joint angle (α) of the thorax-coxa joint, all other leg joints move the leg within the leg plane (orange). The black arrows indicate the directional selectivity of four trochanteral CS groups (G1 to G4) and two tibial CS groups (G6a and G6b) in the stick insect. Owing to their location on the leg and to their physiology, four of these groups signal loading and unloading either within (G3, G4, G6a, G6b) or orthogonal (G1, G2) to the plane of leg movement. For example, G1 responds strongest to posteriorly directed forces. **(B)** Analogous sites for biomimetic strain measurements on the leg of HECTOR. **(C)** Scanning electron micrograph of the trochanter of a stick insect hind leg, with three enlarged sections showing the dorsal trochanteral hair plate (trHP, white circle), and trochanteral CS groups G2 (yellow), G3 (magenta) and G4 (purple). White double-arrows indicate the different preferred strain direction of each sensilla group. **(D)** Putative wiring diagram of known reflex loops involving trochanteral and tibial CS in a standing stick insect, indicating target and sign of the sensory-motor couplings mediated by individual CS groups. Thick lines represent verified sensory-motor couplings, thin lines are presumed on the basis of cursory observations. The schematic motor neuron pools are arranged according to their actions on joints either within the leg plane of movement (orange: levator/depressor system of the Coxa-Trochanter (CTr) joint and flexor/extensor system of the femur-tibia joint) or perpendicular to this plane (yellow: protractor/retractor system). Note that the excitatory connection from tibial CS group G6b to the depressor of the trochantero-femur establishes a muscle synergy through inter-joint coupling within the leg plane. The asterisk at the inhibitory connection of CS group G3 to the depressor indicates that a sign reversal is known to occur during walking.

Taking an evolutionary view, most insect mechanoreceptors are derived from ciliated epithelium tissue. As such, they are either embedded within or immediately attached to the cuticle of the exoskeleton. As the cuticle covers the entire insect body, cuticular mechanoreceptors may be found on all body segments, with particularly high density on legs, wings and feelers. The basic type of these epithelial mechanoreceptors is a tactile hair that is innervated by a single mechanosensory cell (*Sensillum chaeticum*). Groups of such tactile hairs are often located near the joints, forming patches or rows of hairs that may get deflected during movement of the adjacent joint. These hair plates serve as joint angle sensors (Figure 3C). A more derived version of epithelial mechanoreceptor is the *Sensillum campaniformium*, in which only a small cap- or dome-shaped structure can be seen externally. Like hairs, they typically come in groups, as indicated by the yellow, magenta and purple circles in Figure 3C. Campaniform sensilla (CS) are located at strategic locations for monitoring strains in the exoskeleton, usually near the joint at the base of a segment, where skeletal strain may be immediately related to a load imposed to the end of the segment. For example, at the base of the leg, cuticular strains can arise due to: (i) self-generated forces and torques (contraction of proximal leg muscles); (ii) constant body load; (iii) shifts in body load due to altered body orientation and/or slipping of legs; and (vi) externally applied loads. Indeed, the structure that is of particular relevance to load sensing in insects is the trochanter, a short leg segment that, in many insects, is firmly attached to the base of the femur, i.e., the first long leg segment (Figures 3A,C). The trochanter carries a number of proprioceptive organs that signal load and positional information. For example, Figure 3C shows a posture-encoding hair plate (white circle) and three groups of load-encoding CS groups (colored circles).

Because coxa-trochanter (CTr) and femur-tibia joints are hinge joints with nearly parallel joint axes, they cause the leg to move in a plane (see Figure 3A). The high density of CS groups on the trochanter ensures that loads are monitored at the proximal end of this leg plane, where force magnitudes are largest and, hence, resolution is maximal. The CS groups not only reliably encode magnitude and rate of change of force increments and decrements (e.g., Zill et al., 2011), their exact location and orientation in the exoskeleton also make them directionally selective. For example, CS groups G3/G4 are most sensitive to loads applied within the joint plane, whereas CS groups G1/G2 are most sensitive to loads applied perpendicularly to the leg plane. Other CS groups, e.g., G6a/G6b on the base of the tibia, supply further information about loads applied within the leg plane (Figures 3A,C). The tuning curves of these CS groups thus constitute a reference frame of load encoding that is aligned with the movement plane of the leg (Zill et al., 2012).

Owing to this alignment, the reference frame of load encoding is also congruent with the actions of the leg muscles. For example, protractor/retractor muscles of the thorax-coxa joint will cause actions that impose loads in the direction perpendicular to the leg plane. This is monitored by CS groups G1 and G2. Indeed, our current knowledge of the local reflex circuitry in walking legs suggests that each CS group affects the activity of those muscles that may alter their own sensory reading (Figure 3D). For example, activation of individual G4 receptors in a quiescent stick insect induces depressor activity, whereas activation of G3 receptors reduces the activity of that same muscle. In both cases, the resulting change in muscle activity resulted in force changes driving leg movement within the leg plane (for more details on CS activity and muscle synergies, see Zill et al., 2015, 2017).

Whereas this framework of distributed reflexes stabilizes the posture of a standing animal against perturbation, the situation becomes more complicated during locomotion. This is because the reflex effects of a given CS group may reverse during active motion. This is reminiscent of a force enhancement mechanism known from vertebrates (Prochazka et al., 1997a,b; Donelan and Pearson, 2004), where afferences from Golgi tendon organs are involved in a positive force feedback loop. The state-dependent reversal of the motor effects of CS groups G1/G2 on the protractor/retractor muscle system in stick insects suggests a similar mechanism in insect locomotion (Akay et al., 2007; for a similar effect on G3/G4, see Zill et al., 2012).

Figure 3D summarizes the motor effects of load sensors distributed on a stick insect leg. Note that connections indicate the target and sign of a reflex, not necessarily an identified monosynaptic neural connection. In a standing animal, this circuitry constitutes a set of negative feedback loops that may serve to limit excessive forces (Schmitz, 1993; Haberkorn et al., 2019). For example, combined excitement of CS groups G3 and G6a (magenta combination in Figure 3D) might be caused by large depressor forces, acting to push the leg downwards and outwards. The joint inhibition of extensor and depressor muscle activity will reduce the strain sensed by these CS groups on both the femur and the tibia. In active animals, the sign of at least some reflex actions may reverse (e.g., marked by asterisks in Figure 3D). In this case, a depressor force will lead to further enhancement of depressor force, thus forming a positive force feedback loop. This may aid sustaining the body weight during walking.

### Distributed Proprioception in HECTOR

**Figure 4:**
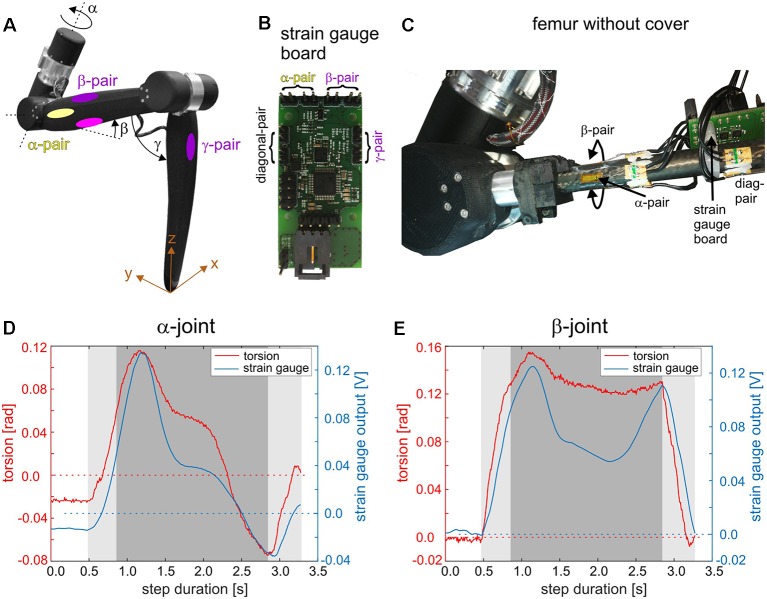
Distributed proprioception in a leg of HECTOR. **(A)** A single leg of HECTOR with elastic α-, β-, and γ-joint drives (equivalent to the thorax-coxa, CTr, and femur-tibia joints of insects). Yellow and purple/magenta ellipses on the femur segment indicate locations for application of strain gauge pairs on the carbon fiber rod under the femur cover. Each strain gauge pair is named according to the joint axis which is predominantly responsible for the bending of the respective pair (colors as in Figure 3). The γ-pair is situated on the tibia segment. **(B)** Small-scale strain gauge board for the processing of four strain gauge pairs. The board can be mounted on the femur segment and connects to all strain gauges used on the leg structure. It can also be connected to the BioFlex bus of the robot communication infrastructure (see Figure 5). **(C)** Femur segment of the leg without cover, revealing the strain gauge pairs glued to the carbon fiber rod. The diagonal pair is sensitive to torsion of the femur and has no obvious biological equivalent. **(D)** Time courses of the α-joint torsion and the corresponding reading of the α-strain gauge pair during a single step. During the experiment, the leg was mounted to a sliding tether that simulated body movement by allowing the leg to push its own base forward and upward during stance (x and z directions in **A**). The dark gray area highlights the time interval during which the leg base was actively lifted and during which the leg had to carry its own weight. The light gray area indicates loading and unloading. **(E)** Same as **(D)** but for β-joint torsion and β-strain gauge pairs.

Given our current knowledge on distributed proprioception in insects and its relevance for adaptive coordination of multiple joints and legs, it is compelling to transfer some of its principles to technical walking machines. Structurally, the carbon-fiber-enhanced, light-weight exoskeleton of HECTOR is well-suited to be equipped with mechanoreceptors at various locations. For example, the principles of distributed load sensing in an insect leg may be mimicked by corresponding pairs of strain gauges placed at the locations indicated in Figure 3B. The matched pairs of loading/unloading-sensitive CS groups, e.g., G6a/G6b (Zill et al., 2011), could be abstracted by a pair of corresponding strain gauges on opposite sides of the same leg segment. This has been done for a single leg of HECTOR as indicated in Figure 4A. All strain gauge pairs are connected to a strain gauge board (Figure 4B) which communicates the strain information *via* the bus system of HECTOR (see below). Figure 4C shows a close-up of the α- and β-pair glued to the carbon fiber rod of the femur.

Figures 4D,E compare the α-torsion of the elastomer coupling in the α-joint drive with the output of the respective α-pair of strain gauges (**D**) and the β-torsion of the elastomer coupling in the β-joint drive with the output of the β-pair of strain gauges (**E**). Results show representative measurements for a single step of the leg which was mounted to a frame that allowed passive sliding of the leg base in the upward and forward direction during stance (gray areas in Figures 4D,E). The results show that both information sources, joint torsion and segmental strain, are analogous to each other but show different temporal response components due to different material properties of the measurement substrate (nitrile rubber in the elastomer coupling; carbon fiber rod at the femur). Strain gauges, however, potentially allow the measurement of strain also in directions which are not picked up by the elastomer couplings.

Irrespective of whether load distribution among legs is measured inside the joint drives or *via* bending forces, several sensor elements need to be read out simultaneously, or at least with similar data acquisition rates. In insects, this requirement is met by the distributed organization of the CNS, where most afferents from sensory organs of a given segment project into the specific ganglion of that body segment. For example, all afferents from trichoid hairs or CS on a middle leg project to the ganglion of the mesothorax. Since each segmental ganglion can be considered a stage of local information processing, including the circuitry for generating motor commands, sensory-motor control is highly distributed and de-centralized (see also “Modularity of Insect Motor Control” section).

**Figure 5:**
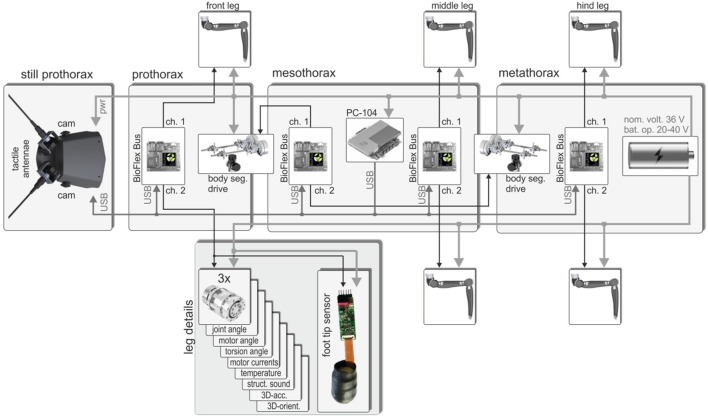
Communication scheme and location of main electronic parts of HECTOR. The robot has three body segments (pro-, meso-, and metathorax), each one of which carries a pair of legs. The front segment looks like a head as it carries eyes (cameras) and antennae (tactile probes) (Hoinville et al., 2014). Each leg comprises three compliant joint drives that communicate with a bus master (BioFlex Bus) in the respective body segment. Each bus master has two channels (2 Mbit/s each) to connect to a maximum of 250 clients which are polled by the bus master to allow real-time operation. The box for the left front leg lists the 12 sensor readings provided by the integrated electronics board of each joint drive and shows the multi-taxel foot tip sensor of a front leg. The bus masters are connected to the host computer (PC/104) in the mesothorax *via* high-speed USB. A fourth bus master in the mesothorax is dedicated to the two spindle drive setups for the inter-segmental joints.

In a robot with multiple limbs, a de-centralized control concept could be implemented in different ways: one extreme would be to assemble a network of multiple de-centralized hardware modules; another extreme would be the use of a single central processing unit running several separate but interacting software modules. In the case of HECTOR, a mix of these concepts has been implemented: a large number of sensors is read out by a set of only three bus master boards, each representing the information node in one body segment (a fourth bus master was integrated for later communication with the body segment drives). At the same time, a single central controller, located in the mesothorax, receives all sensory information from the bus masters and emulates the distributed control network in software. As shown in Figure 5, HECTOR’s main body consists of three segments, each of which carries two legs. The three compliant actuators per leg contain their own controller electronics (Figure 2F), including local sensors of various kinds (see leg details in Figure 5). Using a custom communication protocol that is based on an RS-485 interface in the hardware layer, the wiring in the legs can be reduced considerably (for details, see Schneider et al., 2012). The *BioFlex* bus master connects both legs of that body segment, including all its sensors and actuators, with the central controller. At present, the sensory equipment of each joint of HECTOR comprises eight different sensor types, supplying a total of 12 measurements per joint. These include the joint angle, the torsion of the integrated elastomer coupling, 3D-acceleration and orientation vectors, etc. (see leg details in Figure 5). Potentially, the central controller can thus exploit 216 measurements from the 18 leg joint drives alone, not including strain gauges (Figure 4), foot tip sensors (see “Multi-Taxel Touch Sensor for HECTOR Foot” section), vision or touch (see Hoinville et al., 2014).

The connection between the central controller and the bus masters is realized by USB, thus combining the universal availability of USB with a computationally efficient bus protocol. The efficiency of the bus protocol is especially important for the embedded microcontrollers in the joint drives since they are also responsible for the communication with the motors and must keep up a hard-real-time schedule. Since USB uses differential signaling too, the error rate is very low despite the fact that the communication lines run close to brushless DC motors and power lines. To further reduce the cabling, a common power supply is used for all electronics on board. All segments are supplied with 20–40 V from the battery pack in the rear segment (metathorax in Figure 5).

## Ground Contact and Load-Dependent Coordination

With regard to distributed mechanoreception, the foot is a special case. Because the foot is the main contact zone of the insect body, forces and motions of the foot are immediately related to events occurring at the interface between body and substrate. Even if a considerable part of substrate adhesion may be passive, it is important for animals to detect the onset and offset of ground contact, and to control the muscle forces necessary to achieve, maintain and terminate a firm engagement of the foot with its substrate. In particular when walking on rough terrain with potential step-to-step variation in surface structure and orientation of the substrate, detecting and encoding the properties of ground contact and substrate engagement are essential for postural stability, motion efficiency and, in case of a walking machine, safety.

### Ground Reaction Forces in Insect Walking

**Figure 6:**
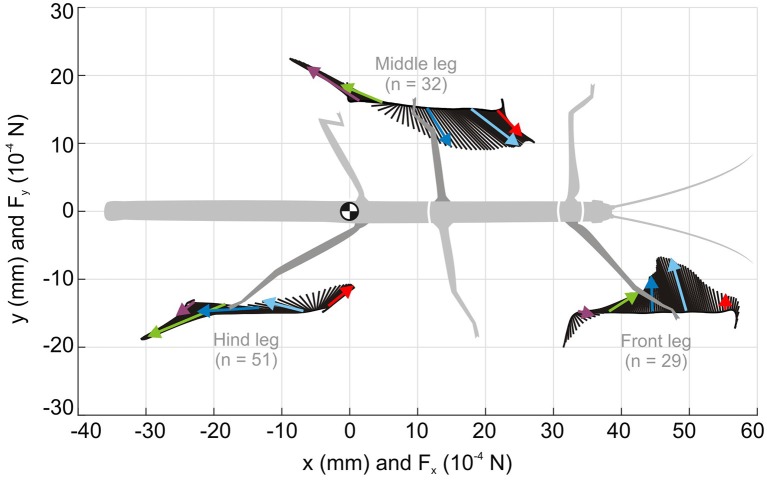
Horizontal ground reaction forces (GRF) during unrestrained locomotion. Average horizontal force vectors of an unrestrained forward walking stick insect on a planar surface (drawn as inverted ground reaction force vectors). The vectors are mapped onto the position trajectories of the respective tarsus in a body-centered coordinate system (origin: the center of mass, being located at the rear end of the metathorax). Data from one representative animal, with separate measurements per leg, normalized to the duration of the stance phase. Black lines show force vectors every 1% of stance duration. Colored arrows indicate magnitude and direction of the horizontal force components at specific times of stance (red: 10%, cyan: 30%, blue: 50%, green: 70%, purple: 90%). Walking direction is from left to right. For details on ground reaction force measurements, see Dallmann et al. (2016).

A look at the forces acting on a foot during a step cycle immediately reveals that “having ground contact” is not a simple binary state, not even when walking on a perfectly flat horizontal surface. For example, Figure 6 shows mean foot trajectories for the stance phases of all leg types in an unrestrained, straight and planar walking stick insect. Additionally, it shows the mean magnitude and direction of the horizontal ground reaction forces (GRF), as measured in the study of Dallmann et al. (2016) at a given time of the normalized stance movement. Clearly, GRF vary strongly throughout the step cycle and differ a lot among leg types. Whereas the “breaking phase” with forward-directed (thus decelerating) force vectors is common to all legs, only middle and hind legs show a clear “propulsion phase” with significant rearward directed force vectors. Inward directed force vectors are also common to all legs, though with different timing and magnitude. In front and middle legs, breaking forces can last up to more than 50% of the stance phase (red and blue vectors in Figure 6). In contrast, a hind leg begins to contribute to propulsion much earlier, i.e., after about 25% of its stance phase (light blue vectors in Figure 6).

Given the fact that a stick insect foot has five tarsomeres, all of which are moved by the same muscle-tendon complex (i.e., with a single degree of freedom for control only), the complex GRF pattern in Figure 6 suggests strong changes in passive forces acting on the foot. Since the tarsomeres are equipped with a variety of mechanoreceptors, including tactile hairs and CS, it is conceivable that they can monitor magnitude and orientation of force vectors as well as size and orientation of the contact area. Indeed, tarsal sensilla have been shown to reliably encode rate and amplitude of loads and resisted muscle forces at the tarsus (Zill et al., 2014, 2017), and to contribute to activation of both the tarsal retractor muscles and the more proximal flexor muscles involved in pulling the leg inward (Zill et al., 2015). This suggests that tarsal sensilla are involved in the establishment and maintenance of substrate engagement.

In addition, signals from tarsal CS could be suitable for detecting increments and decrements of vertical load in the process of triggering transitions from stance to swing (see “Load-Dependent Coordination” section). However, several studies have demonstrated that complete loss of the distal part of a leg does not impair proper step cycle transitions as long as the trochanteral CS groups are intact (Wendler, 1964; Keller et al., 2007). This finding indicates that the CS groups at the base of the leg are sufficient to detect ground contact. Moreover, the sensitivity, orientation, and locations of CS groups 1–4 (Figure 3C) are well-suited to monitor the GRF at the endpoint of the leg. The extensive sensorization of the insect foot could, of course, add more fidelity to the encoding of GRF by trochanteral CS groups. Moreover, it is likely to be relevant for the control of the foothold, in particular for encoding grip force or detecting slip. This is reminiscent of the integration of cutaneous and muscle receptors in vertebrates, where it has been argued that sensory monitoring of the ground contact conditions is relevant for understanding walking and for devising biologically inspired walking models (Frigon and Rossignol, 2006).

### Multi-Taxel Touch Sensor for HECTOR Foot

With regard to our knowledge on foot sensorization in walking insects, the design of a sensorised foot for HECTOR was guided by two main goals: (i) the pressure distribution on the foot tip should be monitored at multiple measurement points, allowing to estimate both the magnitude and the spatial direction of the force vector; and (ii) the sensor array should yield a tactile image of the contact surface, potentially allowing for further analysis and/or classification of the substrate. Once achieved, the combination of these two properties would be of immediate relevance to the use of the foot tip as a sensorised gripper.

For a touch-sensitive foot tip of HECTOR, we chose to use piezo-resistive rubbers because of their smooth dependence of measured resistance on applied pressure (Drimus et al., 2014b). Other important advantages of this material are flexibility, overload robustness and low cost. The material can withstand pressure up to approximately 6 MPa (or 860 psi) for millions of actuations. By using a multiplexing algorithm, we could address multiple sensing elements with a small number of wires. As a result, we could acquire a tactile image by iterating through all possible combinations of matrix columns and rows, yielding a spatial array of measured values at any given moment.

**Figure 7:**
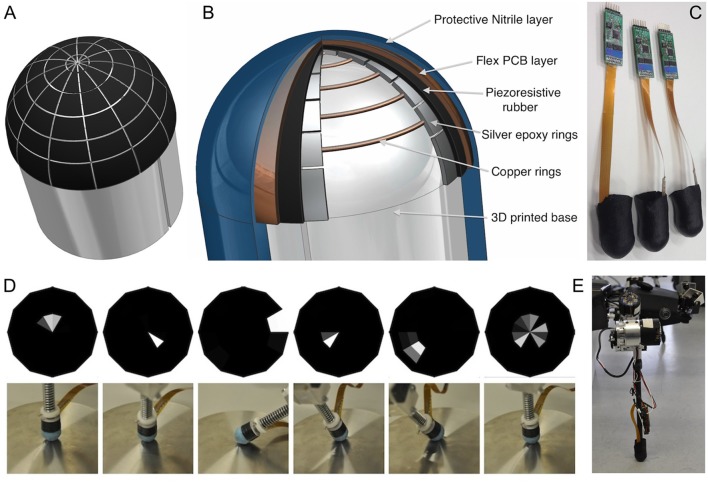
Multi-taxel foot tip sensor. **(A)** Tactile cell distribution across the foot tip surface. There are five concentric rings with 12 sensing cells per ring. **(B)** The section cut through the sensorized foot tip, revealing the layered construction of sensor. **(C)** Finished prototypes with electronics. Lower panel: directional sensitivity of the foot tip sensor. **(D)** Tactile images (top) and corresponding test situations (bottom) for six different end-effector poses. The more slanted the pose, the more marginal is the location of mean activity in the tactile image (adapted from Drimus et al., 2014a, with permission). **(E)** Sensorized end-effector mounted on HECTOR.

A semi-spherical tip covered with as many tactile cells (taxels) as possible ensured that movements of the end-effector were not constrained by the sensor, while contact information could be acquired for most poses. For a foot tip diameter of 2 cm, a radial array structure of 12 sectors and five rings was chosen as the best compromise between manufacturing difficulties, cell size and spatial resolution (see sensor layout in Figure 7B). This determined the spatial resolution as 30° azimuth and 15° elevation for the force direction estimate in polar coordinates, with 60 taxels per tactile image. Among various electrode types, Drimus et al. (2014b) obtained the best results by using* Flex PCB* designs with high-conductivity finish and conductive epoxies (Figure 7). Also, permanent electrical contact between the electrodes and the piezo-resistive rubber patch was avoided, as this reduces the sensitivity for the low-force sensing range.

In order to build a sensor array over a curved surface, we started with a plastic mold of the end effector tip, into which five concentric electrode rings were integrated, that were made of conductive silver epoxy (thickness apporoximately 0.5 mm; 8,331 Silver Conductive Epoxy Adhesive, MGChemicals) yielding a resistivity of 0.017 Ωcm. On top of this bottom layer, we cut a flower-like shape of conductive rubber, uniformly covering the effector tip. The top layer consisted of a custom-developed *Flex PCB* that covered the conductive rubber. With its 12 electrodes, it provided a perpendicular overlap with the epoxy electrodes. Both the epoxy and the *Flex PCB* electrodes were connected to *Flex FFC* connectors over a total of 18 signal wires (ground, 12 top and five bottom electrodes). A final thin protective layer of polyurethane-impregnated textile was applied, not unlike a sock, as shown in Figure 7B. For a detailed description of the manufacturing process, see Drimus et al. (2014a). The finalized prototypes, together with the electronics modules, are shown in Figure 7C.

The basic mechanism for measuring the pressure exerted on each rubber taxel is based on a voltage divider principle as described in Drimus et al. (2014b). Therefore the electronics for data acquisition consisted of a multichannel ADC, multiplexers, power supply and an RS-485 transceiver for integration into HECTOR’s *Bioflex* bus system, along with an Atmel UC3L064 microcontroller. Temporal resolution may be up to 500 tactile images per second. The microcontroller can reply requests *via* the *Bioflex* bus regarding force, pressure or angle estimates, as well as full tactile images with 8-bit values per taxel. According to model calculations, accurate estimates can be obtained for forces as low as 0.1 N. Below that, accuracy deteriorates due to contact resistance uncertainties within the piezo-resistive rubber (Drimus et al., 2014a).

**Figure 8:**
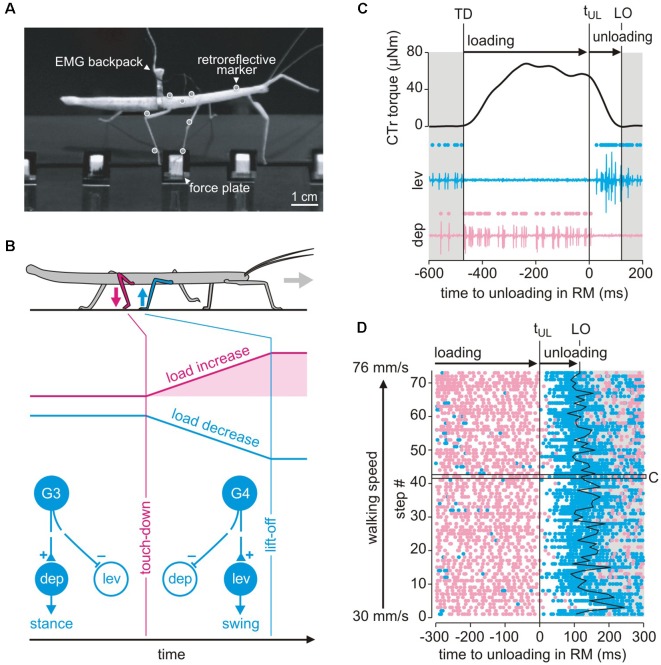
Load-based inter-leg coordination in an insect. **(A)** Experimental setup for the simultaneous measurement of kinematics, GRF and muscle activity. Side view of an animal carrying a light-weight EMG backpack and motion capture markers (white circles) while standing on a force plate with its right middle (RM) leg just as the right hind leg is about to touch down. **(B)** Graphical summary of the putative mechanism underlying the transition from swing to stance in the RM leg after touch-down of the ipsilateral hind leg. CS groups G3 and G4 on the dorsal trochanter are highly sensitive to cuticular strains in the trochantero-femur of that leg. G3 is activated when dorsad bending torques increase, as by loading of the leg during stance. G4 activity signals a decrease of dorsad bending, as during unloading. Schematic of the G3/G4 reflex pathways onto coxal muscles in active animals. Broken lines indicate functional motor effects. G3 afferent activity excites (+) the depressor, i.e., a stance muscle, and inhibits (−) the levator, i.e., a swing muscle (Zill et al., 2012). G4 afferent activity is assumed to have the opposite effect. Unloading induced by a neighboring leg may reverse afferent activity from G3 to G4, thereby promoting the leg’s stance-to-swing transition. **(C)** Unloading of the middle leg coincides with a cessation of depressor activity. CTr torque of the RM leg and simultaneously recorded activity of the levator muscle (blue) and depressor muscle (red) of an example step. Dots above EMG traces indicate muscle spikes detected based on amplitude. TD: touch-down; LO: lift-off; t_UL_: time of unloading. **(D)** Raster plot of detected muscle spikes aligned to t_UL_ of the RM leg (*n* = 73 steps from *N* = 8 animals). Walking speed corresponds to the mean speed of the center of mass during stance. The black box marks the step shown in **(C)**. Note that the depressor activity stops at the time of unloading while levator activation shows a considerable time delay and cannot account for the onset of unloading of the middle leg. This holds true for the entire range of walking speeds tested (adapted from Dallmann et al., 2017, with permission).

The sensorised foot tip was tested by applying forces up to 30 N at different tilt angles, as illustrated in Figure 8D, along with the corresponding tactile images. The results show that the identity of the taxels triggered, as well as the force distribution gives an intuitive estimate of both force magnitude and direction. For incipient contacts (e.g., columns 2 and 4 in Figure 8D), only single taxels are triggered, whereas high contact forces result in the triggering of more cells (e.g., 3rd column in Figure 8D). Previous experiments with similar constructed tactile sensor arrays have shown very good classification rates for palpation procedures with a parallel gripper (Drimus et al., 2014b), as well as classifying different types of cylindrical terrains when used in combination with a compliant robot foot (Borijindakul et al., 2018). The sensorised end-effector mounted on HECTOR is shown in Figure 7E (see also Figure 5). For the presented foot tip sensor, preliminary experiments on substrate classification in response to a vertical contact event were successful for substrates as different as gravel, sand or a solid plane. Furthermore, surface sensing with a flexible leg prototype that was covered with similar piezo-resistive rubber showed promising results in the classification of different types of pipe substrate such as PVC, hard paper and sponge when used in a planar array (Borijindakul et al., 2018).

### Load-Dependent Coordination

Due to their sensitivity and their arrangement at the leg segments, CS lend themselves to monitor distant events, such as lift-off or touch-down events of neighboring legs. The footfall patterns of stick insects reveal a metachronal wave of swing moments from back to front ensuring temporal coordination. Middle legs, for example, start their swing shortly after the touch-down of the ipsilateral hind leg. From behavioral studies (Cruse, 1985) it is known that, besides position parameters, the loading state of the leg is critical for the decision when to switch from stance to swing. Since all legs in stance are mechanically coupled *via* body and ground, the middle leg should be unloaded as the ipsilateral hind leg touches down and starts to take on some body load. In principle, this unloading of the middle leg could be detected by the G3/G4 group of trochanteral CS (Figure 3C).

Given our knowledge of the sensory-motor loops involving trochanteral CS (Figure 3D), one can anticipate that afferent activity from G3 during stance should enhance the activity of the trochanteral depressor muscle, whereas unloading caused by touch-down of the neighboring hind leg terminates G3 activity and leads to G4 activity instead. Afferent activity from G4, in turn, activates the levator motoneurons (Figure 8B). Moreover, recordings of afferent activity from middle legs of free walking cockroaches already suggested that some CS are sensitive to unloading of the middle leg were activated upon touchdown of the neighboring hind leg (Zill et al., 2009, 2012). Using combined motion capture, ground reaction force measurements and parallel electromyographic (EMG recordings of the antagonist levator/depressor muscles of the middle leg in a stick insect (Figure 8A), Dallmann et al. (2017) showed that: (i) the sensitivity of the G3/G4 CS is sufficiently high to sense the torque change at the CTr joint upon unloading of the leg; (ii) the termination of depressor activity coincided with the time of unloading (Figures 8C,D, pink traces); and that (iii) unloading is not due to the onset of levator activity (Figures 8C,D, blue traces). The latter was revealed by the finding that the levator muscle becomes active only with considerable delay to unloading (Figure 8D, white region between pink and blue dots). A model of the animal in static equilibrium allowed the estimation of what may be called the unloading efficacy of a particular leg. Strong differences between unloading efficacy among legs revealed that the ipsilateral hind leg is the most likely candidate for unloading the middle leg with respect to both amplitude and timing (Dallmann et al., 2017). These results indicate that, when a leg touches down on ground during walking, it effectively takes on body load and thus unloads a specific neighboring leg. Given that a leg can detect the unloading reliably, this can locally trigger its transition from stance to swing, thus contributing to temporal coordination of a specific pair of neighboring legs. Since this mechanism of inter-segmental coordination is mediated by the mechanical information flow, it may be a robust, fast, and computationally cheap augmentation to neural mechanisms involving inter-segmental interneurons. Moreover, it is to date the only mechanism that was shown to implement a behavioral coordination rule proposed by Cruse and Schwarze (1988), Cruse (1990), rule 2 in Cruse et al. (1995). As it exploits interaction forces occurring between the legs and the substrate it is an inherently embodied, adaptive control mechanism and, therefore, well-suited for implementation in multi-legged robots.

## Spatial Coordination of Limbs and Omnidirectional Agility

### Spatial Coordination of Limbs in Insects

Recent research on inter-leg coordination in animals has been somewhat biased towards aspects of temporal coordination, to the analysis and modeling of gaits in particular (flies: Wosnitza et al., 2013; Isakov et al., 2016; ants: Wahl et al., 2015; cockroach: Bender et al., 2011; Weihmann et al., 2017; stick insect: Grabowska et al., 2012; Szczecinski et al., 2018). While temporal coordination and its speed-dependent transitions certainly are of general importance to our understanding of steady-state locomotion—particularly regarding considerations of optimality (Weihmann, 2018), it does not account for the control of foot placement. This, however, may be of critical importance for climbing animals. Goats and their relatives provide for extreme examples of this, as they may even climb trees (see Figure 1 in Delibes et al., 2017), and several species inhabit rocky and/or alpine habitats (e.g., Lewinson and Stefanyshyn, 2016) where slipping and falling may cause deadly injuries. In insects, impact-induced injuries will be less critical due to their small mass. Moreover, fast-running insects are known to compensate for mechanical disturbances (Jindrich and Full, 2002) through viscoelastic properties (Dudek and Full, 2006), thus making foot-placement less important. Nevertheless, accurate foot placement will be of behavioral relevance whenever accurate control of limb posture and/or efficient climbing performance will affect fitness, e.g., in foraging, escape or camouflage behaviors.

Given the proprioceptor types of insects (Horridge, 1965; McIver, 1985; Tuthill and Azim, 2018) and their distinct afferent projection regions in the ventral nerve cord (Tsubouchi et al., 2017), it is plausible to assume distinct neural circuits for the control of force and load on the one hand (see sections above) and the control of limb posture on the other. Moreover, the impressive flexibility of motor behavior in insects suggests flexible recruitment of sensory-motor feedback mechanisms as required for a particular behavioral goal (Dürr et al., 2018). Studies on several behavioral paradigms have shown that limb posture may be set by exteroceptive encoding, e.g., through vision or touch, or by proprioceptive encoding. Examples for visual control of limb posture range from attentive behavior such as antennal tracking of visual objects (Honegger, 1981) to turning-related changes in the movement direction of front legs (Dürr and Ebeling, 2005; Rosano and Webb, 2007) and visually guided foot placement or reaching (Niven et al., 2010, 2012) to decision-making in climbing (Pick and Strauss, 2005). Tactually guided foot-placement occurs in stick insects that use their front legs to reach for a location that was touched by the ipsilateral antenna (Schütz and Dürr, 2011). Accurate foot placement in three dimensions through proprioceptive encoding has been shown in freely climbing stick insects (Theunissen et al., 2014), where foot contact locations of a trailing leg are systematically shifted according to the last foot contact of the leading leg (for review, see Dürr et al., 2018).

Compelling evidence that postural cues may strongly affect or even override otherwise rhythmic mechanisms comes from a simple experiment on the stance-to-swing transition in stick insects. In tethered walking stick insects, a single leg may be taken out of the stepping rhythm by placing the foot on a spatially fixed platform, while the other five legs continue coordinated walking. In this case, the position of the platform strongly affects the likelihood of foot lift-off and the re-emergence of rhythmic stepping of the sixth leg (Cruse and Epstein, 1982; see Supplementary Video 1 of Dürr et al., 2004). Other evidence for the relevance of postural cues in the control of stepping comes from goal-directed turning, e.g., in jumping spiders (Land, 1972). Generally, sensory-induced state transitions in stepping have been included in many models of inter-leg coordination in insects (Cruse et al., 1995; Ekeberg et al., 2004) and mammals (Ekeberg and Pearson, 2005) alike, and all of these examples include postural effects (note that postural cues such as leg retraction angle may co-vary strongly with cues related to interaction force, such as the decrease of load during late stance; see “Load-Dependent Coordination” section).

In insects, postural effects are particularly relevant in limb movements that are not mechanically coupled to the movement of other limbs, i.e., whenever the limb is not in contact with the substrate. For example, removal of a proprioceptive hair field on the trochanter strongly affects the height of the swing movement during in unrestrained walking stick insects (Theunissen et al., 2014), as well as the angular range of single-leg searching-movements in stationary animals (Berg et al., 2013). Removal of the same hair fields also raises the likelihood of intermittent searching movements during free walking (Theunissen et al., 2014). This is in line with an artificial neural network model of “apparent sequencing” of swing and searching movements (Dürr, 2001) that assumes that both movements are controlled by the same recurrent network and that the cyclic foot trajectory occurs whenever the swing movement is not interrupted by ground contact (for a detailed discussion of this matter, see Dürr et al., 2018).

Similarly, cyclic grooming of the hind wing in locusts (Berkowitz and Laurent, 1996) can be modulated by shifting a tactile stimulus such that the foot follows stimulus position (Matheson, 1998). Indeed, the lack of a position-dependent transition from one movement pattern to another (Dürr and Matheson, 2003), the robustness of grooming position against changes in load (Matheson and Dürr, 2003) and the strong effect of sensory manipulation on grooming position (Page and Matheson, 2009) indicate that a continuum of cyclic movement patterns, i.e., grooming at various spots on the body surface, is under postural control.

**Figure 9:**
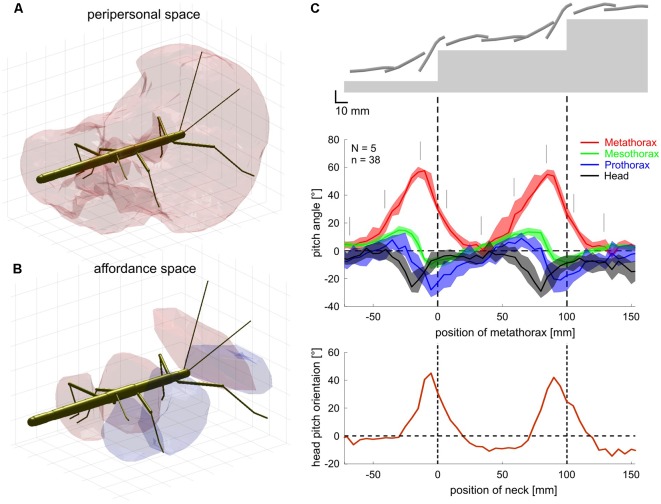
Spatial coordination in insects. **(A)** Active movements of antennae and walking legs of a stick insect delimit a volume around the body within which the limbs may touch an object. Since this volume comprises that part of the ambient space within which motor activity, proprioceptive and tactile sensory input may coincide, it may be called the peripersonal space of an insect. **(B)** Transfer of spatial contact information can work only in those parts of peripersonal space where at least two limbs may reach the same position in space. Since for all such positions, a contact experience of one limb indicates a potential contact position on another limb, an affordance is generated: a limb may reach a contact location on another limb. This was proposed as affordance space by Dürr and Schilling (2018). **(C)** Top: schematic postures of head and thorax as a stick insect climbs a sequence of two stairs. Middle: the inclination of the metathorax changes strongly (red; plotted as a function of metathorax position) and the head and all thorax segments move relative to each other. The head is pitched relative to the prothorax (black), the prothorax relative to the mesothorax (blue) and the mesothorax against the metathorax (green). Gray lines indicate the instants corresponding to the schematic postures above. Bottom: head pitch angle as a function of head position[adapted from Dürr and Schilling, 2018 (**A,B**; CC BY 4.0) and Theunissen et al., 2015 **(C)**, with permission].

Finally, tactually guided, targeted reaching movements of front legs initiate climbing in stick insects (Schütz and Dürr, 2011). In conjunction with the spatial coordination of foot placement between front and middle legs and between middle and hind legs (Theunissen et al., 2014), there appears to be a chain of coordinate transformations from anterior segments to posterior limbs in stick insects. By reaching towards antennal contact locations with the front leg and subsequently placing middle and hind leg feet in very close locations, stick insects appear to exploit prior knowledge of established foot contacts. That way, spatial coordination of ipsilateral limbs can keep locomotion efficient in a variable environment. Such transfer of postural information from one leg to the other can be modeled by a simple feed-forward Artificial Neural Network (ANN, Dean, 1990). This has been exploited in several versions of Walknet, a model of decentralized control of hexapedal walking (Cruse et al., 1995; Dürr et al., 2004; Schilling et al., 2013). Recently, we expanded on the idea of transfer of spatial information among limbs, including antennae and walking legs (Dürr and Schilling, 2018). Based on a large sample of behavioral data, we first determined the size and shape of the volume comprising all positions that are within reach of any limb (Figure 9A). In analogy to the psychophysics of human reaching, this volume was termed the “peripersonal space” of a stick insect. A subspace of peripersonal space was then defined as the set of all foot positions that may be reached by at least two limbs. Within this “affordance space” (Figure 9B) accurate transfer of spatial contact information can be modeled by sets of small feed-forward ANNs (with neuron numbers within a physiologically realistic range; Dürr and Schilling, 2018). With regard to the neural representation of near-range space, these results show that a behaviorally relevant form of representation may not require the existence of a map-like, topological representation of external space, but may be implemented as a simple, direct posture mapping among pairs of limbs instead.

A second important aspect of spatial coordination in insects concerns the thoracic joints. Whereas the three thorax segments are firmly merged in the basic bauplan of several insect orders (e.g., in Diptera and Hymenoptera), it is a characteristic of some others that at least one thorax segment can be moved relative to the others. This may be in favor of agile use of the head in carnivorous staphylinid beetles or snakeflies (Raphidioptera), and/or the agile use of the front legs in mantids and mantispids. In the mentioned cases, only the prothorax appears to be moved actively, while the winged meso- and metathorax are firmly fixed to each other. In contrast, several stick insect species can actively move the meta-mesothorax joint as well (Theunissen et al., 2015). This is likely to improve agility during climbing, e.g., by considerable augmentation of the working range of front legs. Although the mesothorax is very long in stick insects, movement of the meso-methathorax joint hardly displaces the middle legs because they are located at the rear end of the segment and support the metathorax together with the hind legs. Figure 9C shows how the stick insect *Carausius morosus* uses its thorax joints during climbing, where the meta-mesothorax and meso-prothorax joints (Figure 9C, green and blue, respectively) cover mean ranges of 20–30 degrees as the animal climbs a stair approximately three times the body height. Movement of the mesothorax alone thus accounted for an increase of working range of the front leg tarsus by more than 8 mm, equivalent to about 30% of the leg length.

A third major role of spatial coordination is to control the magnitude and direction of the force vector for propulsion, i.e., the net force accelerating the center of mass. Since all joints of the legs in stance are mechanically coupled in parallel closed kinematic chains (at least when assuming no slip of the feet), a torque generated at any joint within this parallel set of closed kinematic chains will affect most (if not all) of the other joints. Whereas in animals this aspect of spatial coordination mainly concerns the efficient coordination of joint torques and, therefore, energy requirement, in engineering it is also a matter of avoiding high tensions that could harm the electronic actuators.

In curve-walking insects, spatial coordination affects the direction of the stance trajectory (Jander, 1985; Jindrich and Full, 1999; Dürr and Ebeling, 2005; Gruhn et al., 2009) and a modification of the spatial coordination of touch-down and lift-off positions between leading and trailing legs (Jindrich and Full, 1999; Ebeling and Dürr, 2006). The associated, transient changes in gait during turning are, at least in part, a consequence of the altered stance directions and step lengths. This view draws support from genetic manipulation experiments on *Drosophila*, showing that the proprioception of interaction forces is crucial for maintaining course (Isakov et al., 2016).

Owing to the distinct control problems for mechanically un-coupled swing, search and/or reaching movements as opposed to mechanically coupled stance movements, several modeling approaches have suggested to treat the two problems with separate control modules (e.g., Cruse et al., 1995; Espenschied et al., 1996). It is important to note that this separation of swing and stance control is mainly a conceptual one, and does not imply these control modules correspond to distinct physiological networks (Dürr et al., 2018). For example, early versions of the distributed neural network controller Walknet suggested a high-pass-filtered positive-feedback mechanism for the coordination of retraction and depression among multiple legs in stance (Cruse et al., 1998) that was inspired by state-dependent reflex reversal from resistance to assistance reflexes (for review, see Pearson, 1995; Büschges and El Manira, 1998).

From an engineering perspective, the adaptive modulation of local reflexes has been applied very early to six-legged (e.g., Berns et al., 1994; Ilg and Berns, 1995) and four-legged (e.g., Albiez et al., 2003) walking machines. In particular, the concept of local positive velocity feedback has been applied successfully for coordinating multiple legs in stance (Schneider et al., 2006). Based on these proofs of principle, it can be concluded that the distributed proprioception and the adaptive modulation of multiple local reflex circuits are sufficient for the control of a multi-legged robot locomotion (Schmitz et al., 2008). As yet, it is a complex problem, requiring either careful tuning or autonomous learning of multiple reflex pathways.

### Omnidirectional Walking in HECTOR

Walking in HECTOR is organized in a computing framework that consists of four main software modules. The actual *walking controller module* was implemented in Python 3 with some time-critical routines like kinematics calculations and stability checks written in C++ and integrated *via* Swig. The *dynamics simulation module* was implemented in C++ using ODE. The *walking controller module* can either be connected to the *dynamics simulation module* or to a *middleware module* (if real robot operation is desired), both *via* TCP/IP. The *middleware module* (implemented in C++) translates control messages from the *walking controller module* and sends the required commands to the bus master boards in the body segments of the robot (see Figure 5). Details of the framework and a flow chart of the overall control sequence for walking can be found in Paskarbeit, 2017 (p. 42 and p. 124).

**Figure 10:**
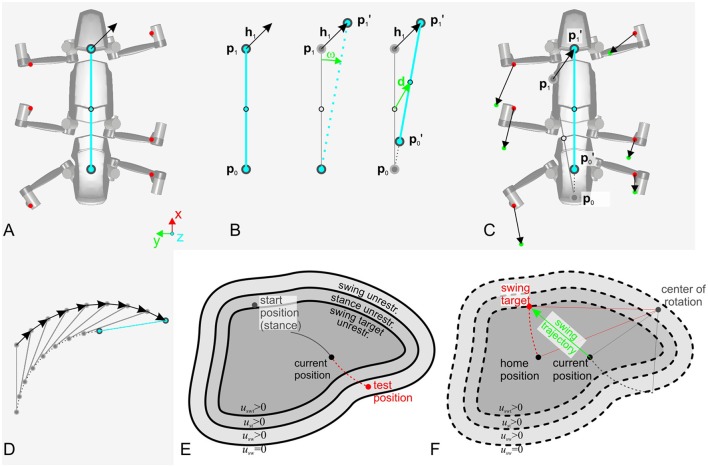
Spatial coordination in HECTOR. To direct the robot into a desired direction, two “pull points” may be used. **(A)** The two pull points, p_0_ and p_1_ are defined on the virtual body midline (blue circles). Foot positions are shown as red dots. **(B)** Concept for the computation of the rotation angle ω and the displacement vector d. Based on these two values, a transformation matrix can be constructed. The inverse of this matrix is applied to the leg tips in order to calculate the leg trajectories for the next time step which is shown in **(C)**. Panel **(D)** indicates the movement of the pull points and the robot midline for a sequence of transformations. **(E)** During the resulting stance movement of a single foot on the ground, the leg must not leave its physically limited working area. In HECTOR this limit is formulated in terms of an unrestrictedness measure. For an ongoing stance movement, the current trajectory is extrapolated beyond the current position, yielding a test point that is checked for its unrestrictedness value. If this value lies below zero, a swing movement is elicited. **(F)** The target of the swing movement is set to a point on the unrestrictedness boundary. It is the intersection point with the backward extension of the current robot movement (red dotted line, attached to the home position of the leg). Instead of explicit transformation matrices, the internal body model may be used as well to estimate the respective movements of the feet.

The walking controller of HECTOR implements distributed control with each leg being considered a separate agent that locally controls the alternation of stance and swing movements. The transitions from stance to swing and* vice versa* are governed by local rules acting between adjacent legs (Cruse, 1990; Cruse et al., 1995). For reasons of robustness, however, the spatial coordination of foot trajectories during turning is not controlled by modulation of distributed reflex loops. Instead, the central directional control of the whole robot is combined with the concept of local leg coordination as illustrated in Figure 10. The movement of the central body axis is considered (blue line in Figure 10A). This axis runs from a point **p**_0_ between the hind legs to a point **p**_1_ between the front legs with a center point in the middle (Figure 10B). These points can be used as “pull points” at which a pull vector **h** can be applied to initiate movement of the central body. By varying **h**, the robot may navigate into a desired direction. The example shown in Figures 10A–C uses only a single pull vector **h**_1_ at the front of the robot. Knowing **h**_1_, one can compute both the rotation angle ω and the displacement vector **d** to describe the intended movement by means of a transformation matrix. Assuming that the legs remain at their position before the displacement but the body is shifted towards the new positions **p**_0_′ and **p**_1_′ the inverse of the transformation matrix can be applied to the foot positions of all legs on the ground to calculate the individual leg trajectories for the next time step of a stance movement (Figure 10C). In the current example, constant application of pull vector **h**_1_ would move the body of the robot as indicated in Figure 10D. As an alternative to the explicit calculation of the inverse transformation matrix, an internal body model as described in “Modularity and the Decentralized Coordination of Multiple Limbs” section may be used for an implicit determination of the stance movements of each leg in the next time step. Since the pull vector **h**_1_ may be oriented in any direction the stance movements of individual legs need no longer be aligned with the fore-aft-axis of the robot as in straight walking or in slight curves.

As a consequence of this framework, the swing-to-stance transition does not take place at a specified posterior extreme position of the leg (see PEP in Cruse et al., 1995). Instead, the stance movement needs to be restricted in any direction with respect to the workspace of the individual leg during omnidirectional walking. The limit of the workspace is formulated in terms of an unrestrictedness measure (Paskarbeit, 2017) which has been derived from the complementary concept of restrictedness as formulated by Fielding and Dunlop (2004). An example for such a limited area is shown in Figure 10E. At the start position of stance, the leg conducts a stance trajectory according to the desired movement of the central body as described above. The course and curvature of the stance trajectory is extrapolated beyond the current position to yield a test point in each control cycle. The test point is then checked for its unrestrictedness value: if the value lies below zero, a swing movement may be elicited, otherwise the leg remains in stance. The target position of a swing movement is set to the intersection point where the backward extension of the last stance trajectory, laid out from a home position of the leg, crosses the unrestrictedness boundary (Figure 10F). This ensures that the leg can continue the last stance movement after touch-down.

**Figure 11:**
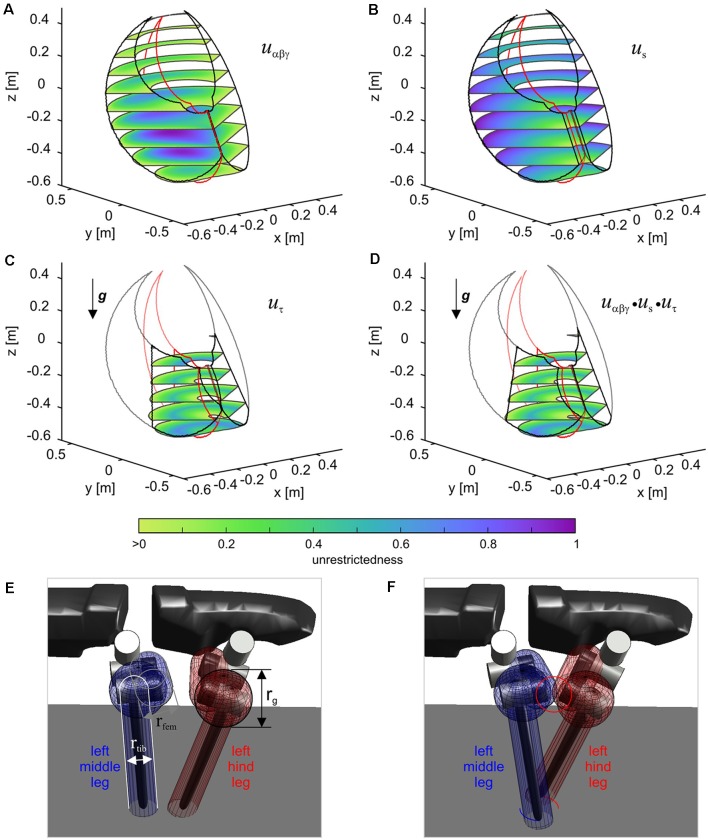
The unrestrictedness measure in HECTOR. Volumetric representation of unrestricedness values for the left middle leg of HECTOR. The horizontal slices are set at distances of 0.1 m in z-direction. Panel **(A)** shows the joint angle unrestrictedness. Black and red contour lines are given for α-angles of 0 and ± 1 rad. **(B,C)** The singularity unrestrictedness **(B)** and the torque unrestrictedness **(C)** for a vertically directed gravity vector. **(D)** Combination (product) of the three unrestrictednes measures of **(A–C)**. Any unrestrictedness value larger than 0 indicates a position which can be reached safely. Panels **(E,F)** show a non-collision and a collision situation between two neighboring legs, respectively. The distance between the enveloping geometric primitives can also be used for a further unrestrictedness measure.

The boundary for each leg results from a projection of a volumetric representation of unrestrictedness. The basic unrestrictedness measure is a scalar value that ranges from zero to one. Any volume in the workspace of a leg which has unrestrictedness values within this numeric interval can be reached by the leg. If a point in space has an unrestrictedness value below zero, it is restricted by definition and should not be entered by the leg. Hence, this can serve as a trigger for a stance-to-swing transition. Note that the conditional definition of the unrestrictedness volume is similar to the affordance volume described in conjunction with Figure 9B, except that there the boundary depends on a condition involving two legs, not just one. Indeed, unrestrictedness values can be described for various aspects of a leg that could potentially restrict leg movement. Figure 11 shows three examples for the left middle leg of HECTOR: the joint angle unrestrictedness u_αβγ_ (Figure 11A), the singularity unrestrictedness u_s_ (Figure 11B) and the torque unrestrictedness u_τ_ (Figure 11C). Since the values run between zero and one, multiple unrestrictedness values can be combined by computing the product (see Figure 11D). In different walking situations, different kinds of unrestrictedness measures may be considered. For instance, the torque unrestrictedness may be neglected during a swing movement, whereas in stance the torque limits of the drives must be maintained. A further useful unrestrictedness measure is the smallest distance between the geometric envelopes of two adjacent legs. As suggested by Figures 11E,F, this may be used to tell collision from non-collision constellations among legs.

Example trajectories of HECTOR’s body segments during curve walking based on this control approach are shown in Figure 13E with the respective podogram in Figure 13F. Here, it becomes evident, that regular gait patterns are exceptions in walking situations with constant heading, speed and environmental conditions. A fixed gait pattern during curve walking is neither necessary in insect walking (Figures 13A,B), nor in robotic walking with moveable body segment joints (Figures 13C,D) or in the control case discussed above (Figures 13E,F).

**Figure 12:**
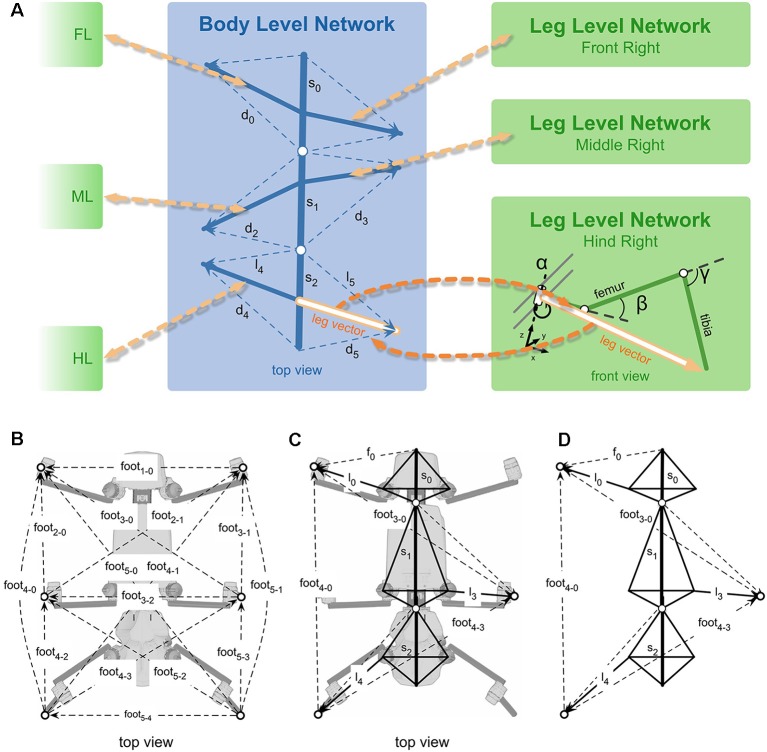
Hierarchical internal body model. **(A)** The body model comprises a body level network (blue) and six subordinate leg level networks (green). Left: the body level network controls the six leg vectors, l_0_ to l_5_, and the three main body segment vectors, s_0_ to s_2_. Right: each one of the six leg level networks controls a leg with three joints and segments. The two levels are inter-connected *via* the shared representation of the leg vector (white arrow) (adapted from Schilling and Cruse, 2012, CC BY 4.0). **(B)** Higher level of the internal body model. Arrows show all constituting vectors that are used to construct the local equations and relationships used to set up an Mean of Multiple Computations (MMC) network for the control of HECTOR. **(C,D)** One specific walking situation of a tripod gait, with the left front and hind leg on the ground together with the RM leg (with and without the underlying robot schematic). During the control of stance only those leg vectors are considered that are interacting with the ground, thus potentially contributing to propulsion, balance and steering. Legs that are currently in swing are suppressed within the body model (adapted from Schilling et al., 2012; © 2012 IEEE).

**Figure 13:**
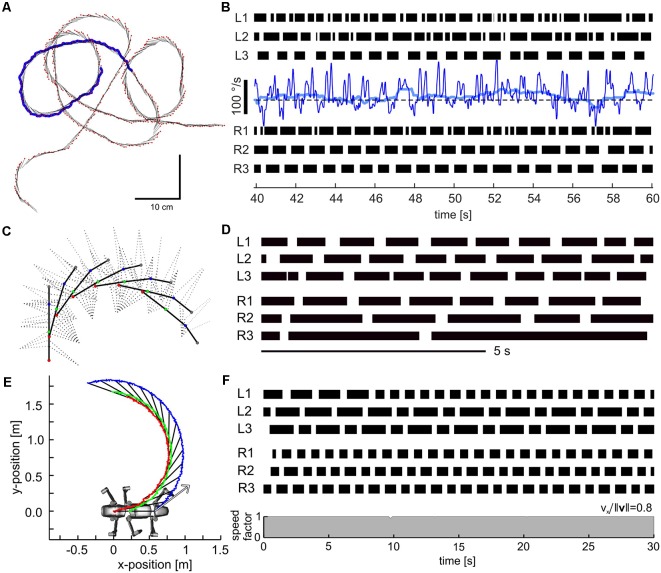
Curve walking in stick insects and HECTOR with and without a body model. **(A)** Sequence of a free walking, blindfolded stick insect on a horizontal plane. Black line segments and red dots show body axis and head every 200 ms (duration: 106 s; median speed was 35 mm/s at the beginning and 25 mm/s at the end). Bold blue line labels the part shown in the podogram. **(B)** Podogram with black lines showing stance episodes of all six legs (L1 to L3: left front to hind legs; R1 to R3: right front to hind legs) and corresponding yaw rotation of the body axis. Blue lines show median rotational velocity per 60 ms window (thin dark blue) and per 1 s window (thick light blue). **(C)** Snapshots of the simulated HECTOR turning to the right using the internal body model of Figure 12. The internal body model was constantly pulled to the front and the right. Snapshots show one body posture per second and four leg postures per second (Figure 8B from Schilling et al., 2013, CC BY 4.0). **(D)** Podogram of the complete run shown corresponds to a turn of about 180°. The lower bar corresponds to 5 s of real time, or 500 iterations of the simulation time (adapted from Figure 7 from Schilling et al., 2013; CC BY 4.0). **(E,F)** Trajectories **(E)** and corresponding podogram **(F)** of the HECTOR simulation using the restrictedness measure as described in Figure 10, but not the body model. Red, green and blue lines in **(E)** show trajectories of the hind, middle and front segment, respectively. Gray arrow shows the pull vector.

## Modularity and the Decentralized Coordination of Multiple Limbs

### Modularity of Insect Motor Control

Despite the importance of central brain structures such as the Central Complex for the selection, control and maintenance of heading (e.g., Strauss, 2002; Neuser et al., 2008; Seelig and Jayaraman, 2013; see also “Conclusions” section), and the significance of small sets of descending interneurons for specific behaviors such as sensory-induced turning (e.g., Zorović and Hedwig, 2013), backward walking (Bidaye et al., 2014) or landing (Ache et al., 2019; for review, see Bidaye et al., 2017), the control of locomotion in insects is highly decentralized. For example, there is no single region or network that governs the execution of a particular gait. Rather, step cycle parameters such as duty factor or stance duration vary continuously with velocity, resulting in a continuum of gaits (for review, see Dürr et al., 2018). Accordingly, there appear to be several network “modules” that interact to give rise to the overall behavior. Anatomically, the modularity of motor control networks in insects is reflected already by the segmental architecture of the CNS, with the ventral nerve cord comprising one ganglion per body segment, connected by nerves that may cover distances of up to several millimeters between the thoracic ganglia (note that thoracic ganglia are fused in more derived taxa such as flies). Each one of the thoracic ganglia contains the complete set of motoneurons that drive the two legs of the corresponding thorax segment.

As a result of pharmacological activation studies, each thoracic ganglion is thought to comprise distinct neural oscillator circuits for different leg joints, thus forming the basis of alternating activity of antagonistic muscles acting on the same joint (Bässler and Büschges, 1998). In stick insects, pharmacological activation appears to induce only little coordination of oscillatory activity among different leg joints (e.g., Büschges et al., 1995), whereas persistent coupling among leg joints has been reported for other insects (e.g., Ryckebusch and Laurent, 1993). Similarly, pharmacologically induced rhythmic antennal movements in stick insects show the same pattern of inter-joint coupling if proprioceptive feedback is still present (Krause et al., 2013). Although the neural components of the local oscillator networks in the walking system of insects remain elusive until today, the idea of coupled oscillators can be applied successfully in modeling of rhythmic intra-leg (e.g., Daun-Gruhn and Tóth, 2011) and inter-leg coordination (e.g., Tóth et al., 2015). As yet, only modeling approaches that emphasize sensory coupling between joints (Ekeberg et al., 2004) and between legs (Szczecinski et al., 2014, 2017) in addition to central oscillator activity can account for insect-like motor flexibility (Dürr et al., 2018). Correspondingly, several robotic approaches that were based on coupled oscillator networks have used sensory input for switching between distinct states in motor behavior (e.g., Ijspeert et al., 2007) or the entrainment of coordinated limb-movements (Owaki et al., 2013) over a range of walking speeds (Owaki and Ishiguro, 2017).

Owing to the distributed and de-centralized organization of proprioception (see “Distributed Proprioception of Posture” and “Load and Ground Contact and Load-Dependent Coordination” sections), inclusion of any proprioceptive feedback adds a “degree of de-centralization.” A purely proprioceptive-feedback driven and, thus, strictly de-centralized approach in the modeling of insect locomotion is Walknet (Cruse et al., 1998; Schilling et al., 2013). This distributed Artificial Neural Network controller implements behaviorally derived rules of inter-leg coordination (Cruse, 1990). To do so, it strictly separates the control of mechanically coupled as opposed to mechanically uncoupled limb movements (Dürr et al., 2004). As a consequence, mechanically uncoupled swing or search movements purely rely on postural feedback (three joint angles per leg, see also “Spatial Coordination of Limbs in Insects” section), whereas mechanically coupled movements are governed by ground contact (postural information is used too, but ground contact causes a switch between control modes). Since Walknet is a kinematic controller, it does not consider interaction forces. In analogy to the considerations of load-dependent inter-leg coordination (see “Load-Dependent Coordination” section), a ground contact signal may be considered a binarized version of an interaction force signal (for further discussion, see Dürr et al., 2018).

Given the considerations about spatial coordination of multiple legs in stance (“Spatial Coordination of Limbs in Insects” section), we propose that sensory information about ground contact or substrate engagement determines the control mode of a given leg. However, as an alternative to the two control schemes of the stance movement discussed earlier, i.e., state-dependent modulation of proprioceptive reflexes and the inverse-kinematics approach described in conjunction with Figure 12A, we introduced an internal, hierarchical body model that can coordinate the movement of all joints which are part of at least one closed kinematic chain. The hierarchical model has been introduced for the control of six-legged walking on flat terrain in dynamic simulation, including negotiating curves (Schilling et al., 2012). This model captured movements of the robot body in two dimensions, only. The model was extended and applied to have the robot HECTOR to climb stairs and to walk across rubble (Paskarbeit et al., 2015). The extension uses a singular-value decomposition approach to control the height of the body (and leg bases) over ground, thus relieving the constraint to two dimensions.

The hierarchical body model approach follows the idea of the passive motion paradigm (Mussa-Ivaldi et al., 1988). It is realized as a recurrent neural network that implements the *Mean of Multiple Computations* (MMC) principle that allows the combined solution of forward and inverse kinematics problems (Steinkühler and Cruse, 1998; Schilling, 2011). MMC networks implement a redundant set of kinematic equations, where each equation describes the triangular geometry of one part of the leg or body. As such, an MMC network maintains the decentralized and modular nature of motor control despite the fact that all equations and, thus, all partial kinematic problems are solved in conjunction by iterating a recurrent neural network. In the following, the basic characteristics of the model will be explained in order to address how this integrates into the embodied control approach.

### A Hierarchical But Decentralized Body Model Based on the MMC Approach

A key principle of an MMC network is that the kinematics constrain the attractor space of the recurrent neural network. Because of these constraints, the activation of the network always corresponds to a correct spatial configuration—or posture—of the modeled body. In a multi-legged agent, considering all possible interactions between the joints that are mechanically coupled at any given instant in time poses a computational problem: the computational effort increases exponentially with the number of joints. In order to reduce the computational complexity of the problem, we proposed a hierarchical body model that allows the distribution of the computational task on two levels (Figure 12A). In this scheme, the lower “leg level” comprises the detailed kinematics of a given leg (green panels in Figure 12A). The higher level or “body level” (blue panel in Figure 12A) comprises the description of the main body segments. In case of HECTOR, the body level comprises the three thorax segments and their relations to the subordinate instantiations of multiple legs. At the body level, there is no detailed information about leg joints. Instead, the leg is represented as a three-dimensional vector that captures the leg’s contribution to support the body. In Figure 12A, this is shown by vectors connecting the main body segments (s_0_ to s_2_) to the feet of the six legs (l_0_ to l_5_), i.e., the ground contact locations. The two levels are connected through shared representations that are present on both levels. This is indicated by the white arrow in Figure 12A. Essentially, this leg target vector “summarizes” the kinematics of the entire leg while, at the body level, it may be regarded as the desired relation between the body and substrate.

On the leg level of the MMC model, each leg is described by a set of three joints and three segments, corresponding to the coxa, trochantero-femur and tibia of an insect leg. As shown in Figure 12A, each leg is described by a kinematic chain with a single degree of freedom per joint, where the α joint sets the orientation of the leg plane, while the β and γ joints move the foot within this leg plane. In HECTOR, as in the stick insect (Figure 3A), the joint axis of the α joint is slanted, causing a change in pronation/supination of the leg plane as the leg is retracted/protracted (see Theunissen et al., 2015; for time courses of this pronation/supination angle, and Dallmann et al., 2016; for consequences on individual joint torque contributions to propulsion and body support).

Other than standard approaches to inverse and forward kinematics of manipulators MMC networks do not suffer the problem of singularities that may prevent finding a suitable and unique solution for the inverse kinematics problem (for details, see Schilling, 2011). This is because for each triangular relation within the MMC network the optimal solution is easily computable. Moreover, multiple equations (one for each triangular relation) contribute to the convergence properties of an MMC network, thus exploiting a redundant description of the body kinematics for computing a mean solution (hence the acronym MMC for *Mean of Multiple Computations*).

In the hierarchical structure shown in Figure 12A, the body and leg levels share the description of the foot positions. At the leg level, this is achieved by describing the posture simultaneously and equivalently by a set of joint angles and by Cartesian coordinates of the foot position relative to the body (along with some mediating diagonal variables). At the body level, each leg with ground contact and all body segments are represented by a vector encoding the foot position. The body model is used differently in the control of swing and stance. As a consequence, only the legs that potentially contribute to propulsion, balance and steering through body-substrate interactions are considered at the upper level of the body model. With regard to the legs in swing, all corresponding equations within the MMC network are disregarded, as if being inhibited. As a result, the equations concerning legs in swing are not taking part in the multiple computations that will determine the posture at the next time step. For example, Figures 12C,D show a typical posture of HECTOR occurring in a tripod gait: only three legs have ground contact at this instant, and only the corresponding three-leg vectors can be used to compute the motion of the parallel closed kinematic chains formed by body, legs and substrate. For determining the posture of the next instant in time, the MMC network implements all possible combinations of connected leg segment vectors, along with additional diagonal vectors describing the interaction of the legs *via* the substrate (Figure 12B). From these, only the vectors shown in Figure 12C are “active” during a tripod stance episode. Much like described for the computations at the leg level, each variable can be computed in multiple ways, using a set of kinematic relations (for details on how to set up these equations, see Schilling et al., 2012).

### Controlling Multiple, Mechanically Coupled Limbs Through a Body Model

Much like what has been described in conjunction with Figure 12A, the control of stance is induced by a passive movement of the front end, as if pulling the body into a given direction. Owing to the recurrent structure of the MMC network, this disturbance of the body model network propagates to all variables contained in the equations for the connected segments. Most notably, this includes all foot positions of the legs in stance. As a result, these variables are adjusted in a way, which complements the enforced movement. Moreover, as foot positions are shared by the body level and the leg level, the induced changes “spread” down into the leg level networks so as to adjust the variables of individual legs. As a consequence, all joint angles of the closed kinematic chains are adjusted in a cooperative way, supporting the overall body movement. The procedure of making these adjustments lasts for multiple iterations, as the network converges into a stable state. Then the resulting leg and segment vectors can be applied to control the actuators. The main difference between the concepts illustrated in Figures 10, 12 concerns the consideration of postural safety in Figure 10, and the simultaneous iteration of all posture control variables in Figure 12.

The body level allows for continuous changes of body orientation. In our simulations and on the robot HECTOR we found that already a single iteration step of the body level is sufficient to come up with good approximations for all leg vectors concerned. When the body level has converged to a particular leg vector, this leg vector serves as an input to the leg level network (Figure 12A, see dashed arrow from left to right connecting the higher with the lower level) ensuing subsequent iterations of these networks and converging to a suitable set of joint angles. In the opposite direction, sensory information acquired by a leg may be used to update the model continuously, thus integrating additional sensory information (Schilling et al., 2012).

The internal body model has been successfully implemented in simulation and on HECTOR. Using dynamic simulations, we first tested the body model in curve walking (Figure 13B; Schilling et al., 2012). It allowed HECTOR to navigate quite narrow curves, with markedly prolonged stance movements of the inner hind leg (R3 in Figure 13B) much like those reported for visually induced tight turns of tethered walking stick insects (Dürr and Ebeling, 2005). When walking slight curves, stick insects show much less asymmetry between inner and outer leg stance durations (Figure 13A) but this strongly increases with increasing curvature of the walked path. As the model simulation was pulled at the front and forced into tight curves, the body level came up with the complementing leg target vectors while exploiting the two inter-segmental drives of HECTOR (see Figure 5). The results suggested that the body model can be used for simultaneous active control of the inter-segmental drives and all legs on ground, allowing for complementary contributions in the negotiation of tight curves. During these simulations, the leg level networks provided robust and stable solutions to the inverse kinematics problems posed by the foot position vector input from the body level. Following the successful application in simulation, the body model has been used on the physical robot as well. There, it has been extended for situations on uneven and rough terrain through a mechanism that adapts the control of body height (Paskarbeit et al., 2015).

### Internal Simulation of Movements and Planning

During its use in the control of stance, the body model essentially serves as a dynamic internal representation of body postures. Its convergence properties allow the flexible use of the same body model to generate appropriate reactive movements to a number of different types of disturbances (e.g., inducing a turn or a change in body clearance). When decoupling the body model from the actual joint drives, the same dynamic internal body model may also be used for movement prediction and planning. Recently, we applied it as an internal simulator to forecast the consequences of different alternative behaviors as a form of planning ahead (Schilling and Cruse, 2017). In this series of simulations, the model served a dual purpose, exploiting its full flexibility in motor control and planning. Besides its application for the control of multiple limbs in stance, as described above, the predictive capabilities of the system were used whenever the system ran into a novel, problematic situation. In a form of trial-and-error search, it was used to test possible behaviors, providing an estimate of their outcomes. These estimates allowed to decide whether the chosen behavior would lead to instability or else might help to overcome the problematic situation. Only if the internal simulation proved successful, the internally simulated behavior was applied to the actuators of the system (Schilling and Cruse, 2017). This shows how an embodied internal model may be grounded in lower-level motor control and can be used flexibly for a cognitive task such as planning ahead (Cruse and Schilling, 2016).

## Internal Models for Body-Size Learning

The relation between the body and the brain is a crucial aspect of embodied robotics (e.g., Nabeshima et al., 2005; Pfeifer et al., 2007). Modern robotic systems are often requested to be very versatile and may even be designed in a completely reconfigurable way. To deal with such complexity, there is a growing demand for simple techniques that allow a robot to autonomously learn the capability of its body without human intervention (Sturm et al., 2008). The MMC model outlined in “Modularity and the Decentralized Coordination of Multiple Limbs” section is based on a predefined set of kinematic equations that is not subject to adaptation or learning at run-time. As a geometric sensory-motor representation of the body, it serves as a body model under the assumption of no growth or damage. In the following section we will consider the plastic use of such an internal body representation in a life-long memory of *Drosophila melanogaster*, implementing a simple form of body model based on recent experimental findings (Krause, 2015; Krause et al., 2019).

### Biological Evidence on Body-Size Learning in Flies

Walking fruit flies can visually estimate the width of a gap in their walkway and engage in energy-consuming climbing behavior only when they see a chance to surmount the chasm (Pick and Strauss, 2005). Since the body size of adult fruit flies depends in part on environmental factors like food quality and temperature during larval stages, there can be considerable size variation among flies of the same genetic background. Therefore, an adaptation process is needed after hatching from the pupal case so that each fly can learn about its own body size. Indeed, visually deprived flies—both freshly hatched flies (Kienitz, 2010) and flies reared in a featureless environment (Krause, 2015)—try to surmount insurmountably wide gaps, whereas flies kept in structured environments with light later take decisions adapted to their body size (Krause et al., 2019).

**Figure 14:**
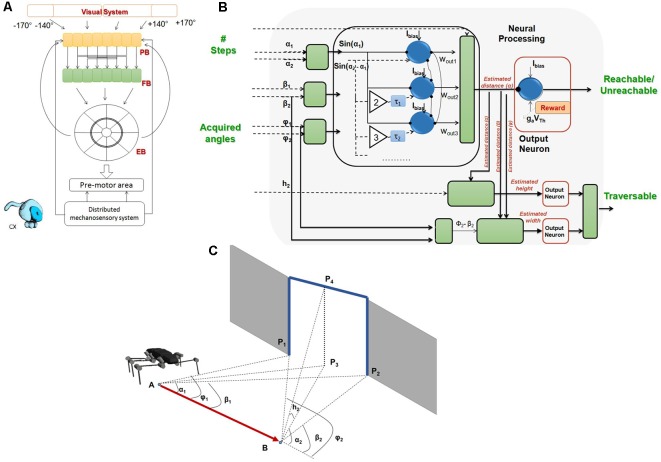
Navigation control structure inspired by the *Drosophila* Central Complex. **(A)** Block scheme of the control structure. The visual system transfers information to the protocerebral bridge (PB) that is involved in body-size learning. The fan-shaped body (FB) participates in visual learning and orientation control whereas the ellipsoid body (EB) is in charge of spatial memory formation. **(B)** Scheme of the network devoted to learning whether an object is reachable within a certain number of steps and whether it is traversable depending on the acquired visual inputs. The green boxes represent simple mathematical transformations, whereas the blue circles represent spiking neurons. The learning process is performed at the level of the output neurons (red boxes). **(C)** Scheme of the angular positions acquired by the visual system as the robot moves forward from point **(A)** to point **(B)**. The object of interest is a gate where different points of interest are detected. Different angles are acquired from the visual system to be processed by the network in **(B)** (adapted by permission from Patané et al., 2018 © 2018).

Experienced small flies abandon attempts on gaps that their larger siblings from the same vials still attempt to climb. The default state of freshly hatched flies before calibrating their size memory is “very large.” They calibrate their body-size estimate to their actual body size by gathering visual feed-back from the retinal images of contrast edges during normal locomotion (parallax motion). The act of physically climbing across gaps is not required for this calibration process. Neurogenetic manipulation revealed that body-size learning requires the cAMP cascade. Learning mutants of the cAMP cascade do not adapt to their individual size. Instead, they try to overcome chasms, which are clearly impossible to cross. Mutant analysis and differential rescue experiments *via* the GAL4/UAS-system revealed that the information is stored in projection systems of the protocerebral bridge (PB) of the central complex (CX, Figure 14A). Furthermore, we have identified the biochemical learning cascade for this life-long body size memory, but the neural circuitry remains to be determined.

### A Computational Model for Body-Size Learning

To simulate the neurobiological findings on gap-climbing *Drosophila melanogaster* flies, we developed a spiking neural network model for body-size learning using parallax-motion information. The model has been implemented and evaluated in a dynamic simulation of HECTOR navigating through a multi-chamber environment. HECTOR has a number of properties that make it a perfect platform for implementing cognitive functions that require embodiment with distributed, multimodal sensory information. One can make use of the embedded distributed sensory system consisting of six pressure sensors located in the tip of each leg, a complete inertial module on the main body and an omnidirectional vision system used to extract the relevant information from the objects located in the environment.

Following the neural structure of the fly brain, the relevant neural assemblies that constitute the Central Complex model are shown in Figure 14A. A neuronal lattice captures the essence of the visual system and is used to acquire spatial information about angular directions of the objects of interest and the heading of the robot. This visual information is transferred to the PB and the Fan-shaped Body (FB), which extract the *where* and *what* for heading control and visual learning, respectively. Moreover, it is mediated to the Ellipsoid Body (EB) for the formation of spatial memory (Neuser et al., 2008; Kuntz et al., 2017). Previous studies have tested this model design in the context of direction control, spatial memory and other capabilities (Soto et al., 2009; Arena et al., 2013b). Here, we report its extension to include the formation of body-size knowledge.

Within the dynamic simulation environment, the robot walks around and detects the position of visible objects of interest. The angular position of an object is acquired through a uniformly distributed ring of neurons that have a one-to-one match with the ommatidia of the eye (about 4.8° opening angle each) distributed in a range of about 300°. The output of the stimulated neuron is modulated with a post-synaptic weight that corresponds to the sinusoidal function of the angular position of the neuron. After forward motion from point A to B as shown in Figure 14C (equivalent to four robot steps in the experiments described here), the robot evaluates the new angular position of the object of interest. This second acquisition is used to estimate the distance between the robot and the object through parallax, i.e., the angular difference in the position of the same object from two different viewpoints.

The distance between the robot and the object is directly proportional to the distance traveled during the parallax-motion estimation, and the coefficient of proportionality depends on the initial acquired angle and its variation when acquired afterward. Starting from this mathematical formulation, a spiking neural network has been designed and implemented to yield similar results (Arena et al., 2013a). A block scheme of the proposed model is shown in Figure 14B. The information about object position acquired in two different time steps is discretized and weighted. An array of Class I Izhikevich neurons (Izhikevich, 2004) is then used to evaluate the ratio between the two acquired sinusoidally modulated inputs.

An array of synaptic gains is used to find the correct match: in the end, excitatory inputs should compensate the inhibitory ones in order to allow the neuron to fire. A bias current was added, making each neuron able to fire with a minimal additional input current. A series of time delays (*τ*_i_) was included to evaluate the neuronal responses in sequence. Each neuron is connected with the others through inhibitory synapses forming a winner-takes-all network topology. The first active neuron (i.e., winning neuron) strongly inhibits the others and produces an output that is proportional to its corresponding gain factor. Assuming that the system knows the distance traveled between the two instants of acquisition (e.g., in terms of number of steps), the outcome of the first part of the network is a signal that is proportional to the estimated object distance. The last processing stage consists of an output neuron that is subject to a threshold adaptation learning process. Depending on an internally generated reward signal, the threshold level is adapted to either facilitate or reduce the spike rate of the neuron. Threshold adaptation can be considered a consequence of the nonlinearities present in the membrane dynamics of a neuron (Izhikevich, 2004). The threshold adaptation process can be modeled as a voltage-dependent current that is introduced as an additional input to the decision neuron. It can be expressed as I_A_ = −g_A_*V_Th_, defining g_A_ as an activation conductance and V_Th_ as a dynamic threshold that is being learned. The current can be modified to hyperpolarize or depolarize neurons. The output neuron thus acts as a gate: its firing indicates that an object is reachable, whereas a silent state corresponds to unreachable ones. Therefore, the decision neuron will provide a prediction of reachability or unreachability that has to be verified by the robot.

At the beginning of an experiment, every object is assumed to be reachable, and in each trial, the robot walks towards a selected target. A reward is generated if the object can be reached within twelve steps. This distance may be considered the maximum energy or time available to fulfil the task. The threshold is then modified depending on the coherence between the reinforcement signal and the internal prediction that is provided by the network. If the prediction is correct, the threshold V_Th_ remains unchanged. Otherwise, if the robot’s assumption of reachability (or unreachability) is not confirmed during the approach, the threshold V_Th_ is increased (or decreased) by a value ΔV_Th_ so as to hyperpolarize (or depolarize) the output neuron accordingly. As the entity of the threshold variation represents a compromise between the speed of the learning process and the precision required, the learning phase will end as soon as the threshold value reaches a steady state condition.

**Figure 15:**
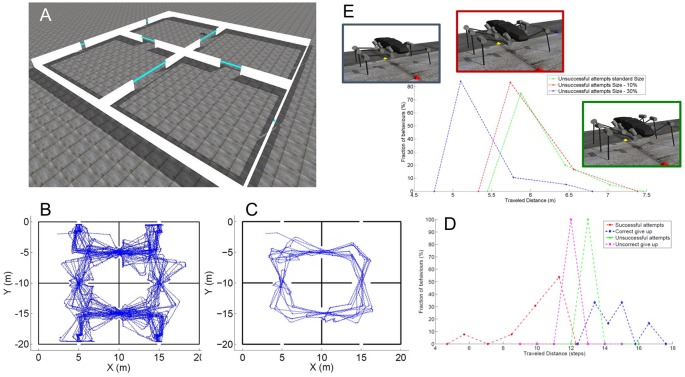
Demonstration of body-size learning. **(A)** The simulation environment consists of four rooms, each one containing three potential passages of different width and height. Each room is 10 × 10 m^2^, the gates facing outside are too small to be passed (width: 0.6–0.8 m, height: 0.3–0.4 m) whereas the other gates are large enough (width: 1.5–2.4 m, height: 0.7–1.0 m). **(B)** Trajectory walked by the simulated robot while exploring the environment during the learning phase. During the learning phase, the robot acquires the knowledge needed for the formation of the internal body-size model. **(C)** Trajectory walked by the robot during the test phase. The body-size model is now used to select and pass the suitable gates while avoiding the others. **(D)** Distribution of the possible behavioral choices made on the basis of the distance output neuron. When the traveled distance is next to the maximum reachable value (i.e. 12 steps) the robot, as in the biological counterpart, can either try to attempt unsuccessfully or to give up incorrectly. **(E)** Comparison between the unsuccessful attempts in three simulations, where the HECTOR model was changed by reducing the tibia segment by 10% (red) and 30% (blue).

Further details on the mathematical description of the network and learning process can also be retrieved in Arena et al. (2013a, 2018), where the proposed network was applied to learn the reachability space in roving and walking robots. In applying this control structure to HECTOR, we adopted the same paradigm for the network structure and the learning algorithm. Through acquiring further sensory information from the visual system, body size learning could be extended to include additional capabilities, allowing the robot to estimate not only the distance of an object but also its height and the width through the selection of points of interest (e.g., center of mass, vertex and others). This was exploited in the experimental test of body-size learning, where the size of gates had to be judged depending on the learned body size (Figure 14C). The scenario of the simulated environment is shown in Figure 15A. It consists of four rooms that are connected by gates of different sizes. At the beginning of the learning phase, the robot was placed in one of these rooms and started to evaluate the estimated distance, height and width of the gates through parallax motion. In a sequence of approaches, the robot first chooses a particular gate at random and then tries to traverse it. Depending on the success of the approach, a reward signal is triggered to adapt the threshold V_Th_ of the output neuron accordingly, thereby tuning the internal body-size representation. Traversable passages were placed between two adjacent rooms, while all the other gates were too small to be used. Therefore, the robot was confined to the four rooms (Figure 15A) while allowing for continuous, autonomous learning. Figures 15B,C show a typical walking trajectory of the robot while exploring the environment.

Since the distance of the robot to the gates varies, the robot opts for different behavioral choices depending on the capabilities of its own body. Figure 15D shows the percentages of selected behaviors depending on the real distance between the robot and the gate. It can be noticed that for low distance values the chosen behavior is an attempt to reach the gate, and the result matches the hypothesis. For distances longer than the reachability threshold, the robot performs a correct give-up (i.e., a change of “interest” in favor of other objects), whereas in the intermediate region, next to the critical distance value, a series of unsuccessful attempts and incorrect give-ups are performed in accord with *Drosophila* experiments (Krause, 2015; Krause et al., 2019).

When the learning process of the output-neuron threshold converges to a stable solution, the robot can use the learned body-size model in the decision-making process. The robot is now capable of identifying gates that can be reached and traversed without getting stuck in a passage that is too narrow to pass. The trajectory performed by the robot at the end of the learning phase is shown in Figure 15C. After learning, attempts to pass through not traversable gates are absent.

To evaluate how the body-size knowledge is related to the actual robot size, the simulated HECTOR robot was modified by shortening the tibia of each leg, thus reducing the “reachability threshold” for a given number of steps. This was done for both a 10% and 30% length reduction of the tibia. The results are shown in Figure 15E, indicating that each robot learns different body-size models, according to the leg length and the corresponding reachability threshold. The maximum number of unsuccessful attempts differs according to the reachability threshold, such that the distribution peaks of the robots with shortened tibiae occurred at shorter distances. In summary, we could show that a spiking neural network model inspired by the *Drosophila* central complex can learn the body size of the robot through interaction with the environment, and in particular by comparing self-generated estimates of working range with the actual behavioral performance.

## Conclusions

The sections “Muscles and Compliant Actuation,” “Distributed Proprioception of Posture and Load,” “Ground Contact and Load-Dependent Coordination,” “Spatial Coordination of Limbs and Omnidirectional Agility,” and “Modularity and the Decentralized Coordination of Multiple Limbs” provided an overview of the potential for integration of multiple lines of research on a common robotic research platform for biomimetic motor behavior, ranging from compliant actuation to cognitive functions. It has been proposed in the past that the goal of biomimetic robots is to “take inspiration from biological principles to design robots that match the agility of animals, and to use robots as scientific tools to investigate animal adaptive behavior” (Ijspeert, 2014). Here, we argue that a further, equally important goal is *to collate and combine biomimetics research on disparate and conceptually disjunct research areas in the neurosciences and engineering sciences in order to integrate insights and concepts on a common platform*. An important step in this direction was initiated with the *iCub* platform of the Italian Institute of Technology, that was introduced as an open platform for research on cognitive robotics, the role of embodiment for cognitive functions in particular (Metta et al., 2008, 2010). Regarding shared research on robot locomotion, a similarly prominent initiative was centered around the quadruped walking robot *LittleDog* of the company Boston Dynamics. Much like *iCub*, *LittleDog* was used by a number of labs to conduct research on the same platform (Murphy et al., 2010). In both of these cases, the research was mainly focusing on computer science topics in cognitive robotics (in case of iCub) or robust controller software for adaptive locomotion (in case of *LittleDog*). Arguably, the outcome of these very successful research networks was mainly in engineering (e.g., cognitive robotics, robot control).

In the case of the hexapod walking robot HECTOR, three properties proved to be particularly important for integrative research. The first of these properties is the highly decentralized hardware architecture (Figure 5) that allows to read out and combine a large number of measurements from different clients (“Distributed Proprioception in HECTOR” section). In the examples provided above, these include 18 sensorised actuators with twelve sensor readings per motor (“The Compliant Joint Drives of HECTOR” section), up to three strain sensor clients (“Distributed Proprioception in HECTOR” section) and one multi-taxel foot tip (“Multi-Taxel Touch Sensor for HECTOR Foot” section) per leg. Together with the second property, the room for additional components inside the exoskeleton (see Figure 1D), the decentralized hardware architecture allowed inclusion of a hardware-accelerated vision system (Meyer et al., 2016) or the use of the prothorax segment as a “head unit” hosting a visuo-tactile system. Finally, the availability of a dynamic simulation environment for HECTOR has allowed researchers from different labs to develop and test components while simulating the use of HECTOR’s hardware properties. To this end, we have concentrated on research regarding three overarching topics in biomimetic locomotion: (i) the particular significance of distributed load sensing; (ii) the emergence of gaits from local coordination rules (or constraints); and (iii) the formation and exploitation of internal representations of body posture and size.

### Load vs. Posture

Three essential variables need to be controlled in legged locomotion: propulsion, stability and heading. All three of these control variables concern the appropriate acceleration of the center of mass (CoM), which, in turn, implies the generation of appropriate forces and torques causing the desired acceleration. Given the physical limitations of the body and its legs, propulsion, stability and heading can only be maintained through coordinated interaction of the limbs with the substrate. At first sight, monitoring the force/torque distribution across the joints of the limbs, and particularly the interaction forces acting on the feet appears to be the most direct way of controlling CoM accelerations. As yet, the effect of a change in torque at a particular joint drive on the CoM can be predicted only, if: (i) the posture of the limb that contains this particular drive is known; (ii) foot contact is sufficiently firm to transmit forces to the substrate without slip; and (iii) all other legs in ground contact give way appropriately in order not to counteract the intended effect. As a consequence, any controller that is to coordinate the movement of multiple legs during stance has to take account of the current body posture. In HECTOR, the measurement of individual joint torques is possible (“The Compliant Joint Drives of HECTOR” section). The three conditions for estimating effects of single drive torques on the CoM can be met by: (i) joint angle readings from the joint drives (“The Compliant Joint Drives of HECTOR” section) and/or the use of a kinematic internal body model (“Controlling Multiple, Mechanically Coupled Limbs Through a Body Model” section); by (ii) monitoring interaction forces with a multi-taxel foot tip sensor (“Multi-Taxel Touch Sensor for HECTOR Foot” section); and by (iii) monitoring strain forces on leg segments (“Distributed Proprioception in HECTOR” section) and a range of control concepts as discussed in “Omnidirectional Walking in HECTOR” and “Controlling Multiple, Mechanically Coupled Limbs Through a Body Model” sections.

Regarding the various sources of distributed sensory feedback that are available during locomotion, recent findings on freely walking stick insects suggest that load-sensing may be beneficial to monitor load transfer among legs and, thus, to determine the appropriate time for a stance-to-swing transition (Dallmann et al., 2017). Similarly, distributed monitoring of load signals have been used successfully for temporal coordination of multiple legs in robots (e.g., Owaki et al., 2013; Owaki and Ishiguro, 2017). Moreover, the normal and tangential components of the ground reaction force vector as experienced (or measured) by an animal provide an immediate link between stability and propulsion. Accordingly, freely walking stick insects adjust the relative activation of antagonist muscles according to altered load distributions when walking on slopes (Dallmann et al., 2019). Finally, the high spatial resolution of a sensorised foot tip can help to extract detailed contact patterns per foot and potentially serve to judge substrate properties (Borijindakul et al., 2018), thus linking locomotion and near-range exploration (for further discussion of this issue see Dürr et al., 2018).

Despite the multiple potential use of distributed force and load measurements, it remains to be shown whether and how insects integrate these distributed measurements for a global control of CoM acceleration. To date, several experimental results suggest that force/load measurements are mainly used for local control, i.e., for assistance and resistance reflexes at single joints (e.g., Akay et al., 2007; for review, see Zill et al., 2004) and, probably, to support temporal coordination of neighboring legs (Dallmann et al., 2017). Recently, it was shown that postural variables stay remarkably unaffected in stick insects that walk up or down steep slopes, despite the fact that this required substantial adjustment of single-joint torques (Dallmann et al., 2019). This suggests that stick insects tend to adjust muscle force so as to maintain a preferred body posture, rather than to adjust body posture so as to optimize force transfer to the substrate.

### Gaits

Unlike in many other walking robots, the gait of HECTOR is not pre-programmed or governed by coupled central oscillators. Instead, the gait emerges from a combination of sensory-motor feedback that regulates limb posture, and/or pairwise coupling of neighboring legs through coordination rules (Cruse, 1990; Cruse et al., 1995). Owing to this approach, a persistent rhythm, i.e., one that characterizes a particular gait, can emerge only once the system enters a steady state. In contrast, transitions in speed, attitude, posture or direction are marked by discontinuities. The most basic types of discontinuity in legged locomotion are the local destabilizing and stabilizing transitions from stance to swing (lift-off) and swing to stance (touch-down), respectively (Dürr et al., 2004). In steady-state locomotion, these step-to-step “local discontinuities” define the overall rhythm or gait. Moreover, they effectively gate the information flow from load and force sensors. This is because strain-sensitive campaniform sensilla afferents of insects fire only if muscle forces are resisted, e.g., during stance (Zill et al., 2012).

In this context, it is important to decide on the function of a swing movement. In the most simple case (and common case in robotics), swing movements are but return strokes of the limb that serve to execute the next stance movement in very much the same way as the preceding one. Essentially, this reduces the control of a swing movement to the inversion of action at every single joint upon lift-off, and a delayed depression in order to re-gain ground contact. In walking insects, however, touch-down locations appear to be under postural control (e.g., Cruse, 1979; Theunissen et al., 2014), and on-going swing movements may be “re-targeted” towards locations detected by the visual (e.g., Niven et al., 2010) or tactile systems (Schütz and Dürr, 2011). Although the latter findings do not concern the stance-to-swing transition, they raise the question as to whether “global discontinuities” such as changes in body inclination or heading could be initiated by swing movements, or need to be initiated during stance. This is not clear because a targeted swing movement and the ensuing altered touch-down location could initiate a new “pulling direction” of the respective leg and, thus, affect the overall acceleration of the CoM during the subsequent stance movement.

In stick insects, the timing of various kinematic parameters suggests that the initiation of visually-induced turning occurs by a change in stance direction of the front legs, both in response to large-field visual cues (Dürr and Ebeling, 2005) and in turning responses towards visual landmarks (Rosano and Webb, 2007). At the same time, the persistent timing of yaw rotation velocity and stance movements of the hind legs during sustained curve walking (Figure 13A) indicates that in turning stick insects the function of the legs may differ between initiating (by front legs) and maintaining rotation (by hind legs). Similar to stick insects, HECTOR translates an intended change of heading into appropriate changes in foot trajectories, either by “global” use of an internal body model that moderates the transitions among the participating legs in stance (Figure 13B), or by “local” use of inverse kinematics (Figure 13C). In both cases, the foot trajectories during stance are terminated depending on postural cues (Figure 12; “Controlling Multiple, Mechanically Coupled Limbs Through a Body Model” section) and/or the combination of various posture and torque measures into a single estimator of unrestrictedness (Figure 11; “Omnidirectional Walking in HECTOR” section). As a consequence, the timing of lift-off very much depends on posture, thus mixing issues of spatial and temporal coordination in the resulting gait.

Therefore, we argue that gaits should be considered a matter of optimality of steady-state locomotion, rather than a matter of control. This view gains support from theoretical considerations of optimal phase shifts in multi-legged locomotion, so as to minimize energy by restraining vertical oscillations of the CoM (Weihmann, 2018). For closed-loop control of locomotion, a fixed gait imposes limitations that are undesirable for locomotion engineering, and probably inefficient for animal locomotion in a variable environment. Accordingly, we propose that gaits should emerge from a control scheme that ensures not only efficient propulsion and stability, but also sufficient adaptiveness in the face of step-to-step changes in body-substrate interaction, and flexibility in the function of particular limbs as behavioral goals change (Dürr et al., 2018). In HECTOR, we achieve this by a combination of de-centralized control and loose, pairwise coupling among limbs through coordination rules and/or the inclusion of internal spatial representations.

### Multiple Spatial Representations

In this study, three kinds of internal spatial representations were considered. Perhaps the most simple form concerned postural mappings between neighboring limbs (“Spatial Coordination of Limbs in Insects” section). This pairwise mapping was originally proposed to model spatial targeting of touch-down locations in ipsilateral pairs of legs by (Dean, 1990), and introduced into the de-centralized walking controller Walknet (Cruse et al., 1995). Such mappings may serve as a distributed representation of the “space within reach” for at least two limbs. The corresponding pairwise posture mappings do not encode spatial information as such, but may serve to transfer spatial contact information to a neighboring limb in a behaviorally relevant manner. As a result, each posture mapping defines an affordance volume (Figure 9) within which a receiver leg can exploit the prior experience of a sender leg (Dürr and Schilling, 2018).

An extension of this approach implements a recurrent neural network to combine postural information about all limbs for the coordination of legs in stance. This was originally proposed by Kindermann and Cruse (2002) and later formulated in a more versatile form by Schilling (2011). The architecture of so-called MMC networks incorporates the geometric constraints of many joint positions in a redundant manner, and converges on solutions that meet these constraints, given a nearly arbitrary set of sensory inputs (see “Controlling Multiple, Mechanically Coupled Limbs Through a Body Model” section). The MMC architecture is not grounded on particular properties of physiological neural networks, except that it uses distributed proprioceptive input and implements recurrent neural connections. A possible neurobiological interpretation of its ability to coordinate the kinematics of parallel closed kinematic chains is that recurrent neural networks with rich proprioceptive input and appropriate connectivity may converge on stable states that should be considered an internal representation of body posture.

Finally, a spiking neural network was proposed that can exploit the consequences of own body actions to form an internal representation of body size (“Internal Models for Body-Size Learning” section). The model takes inspiration from findings on the central complex of the fruit fly *Drosophila melanogaster* (Strauss, 2002; Krause et al., 2019). So far, it is the only part of the described HECTOR project that is devoted to online learning. In essence, it deals with the problem that any internal spatial representation needs to be calibrated to the overall body size or limb proportions (for example, a simulated HECTOR with shorter or longer legs; Figure 15D). In more general terms, whenever body morphology changes during life-time, or cannot be known at a time suitable for pre-programming, there will always be the need to scale internal mappings.

### Integrative Biomimetics

As most other biomimetic robots, HECTOR is not a scaled hardware model of any particular animal species, despite the fact that its original design was inspired by thorax and leg proportions of a stick insect (Figure 1). As outlined in all sections above, the technical issues tackled have their counterparts in experimental neuroscience, despite the fact that none of the implementations on HECTOR come even close to being a one-to-one model of the biological counterpart. The proposed solutions are thus conceptual models that reflect system properties of their biological counterpart rather than their physiological implementation. With regard to integration of different lines of research, we find that it can be advantageous to combine conceptual models because individual subsystems do not have to be adjusted to the particular implementations of other subsystems, as long as they can be interfaced. In relation to Marr’s three levels of analysis (Marr and Poggio, 1976), this loosely corresponds to an integration at the algorithmic level.

Moreover, the different methodologies that were applied in the neurobiological experiments conducted in conjunction with this article (e.g., reflex circuits in Figure 3, ground reaction forces in Figure 6, muscle activity in Figure 8, behavioral physiology of unrestrained locomotion in Figure 9, and neurogenetics of higher-order motor behavior in Figure 14) are paralleled by equally different methodologies in the engineering developments (e.g., mechatronics in Figure 2; sensor technology in Figure 7, modeling of recurrent neural networks in Figure 12, and of spiking neural networks in Figure 14). Since all of these methodologies—both in neuroscience and in engineering—require very different and potentially disjunct areas of expertise, we believe that it not only takes collaborative effort of different research labs to bring these areas of expertise together but that it is absolutely essential to have a common research integration platform like HECTOR and a corresponding dynamic simulation environment to do so.

## Author Contributions

VD, PA, HC, AD, MSchi, JS, RS, and AS: conceived the project. VD, PA, RS, and AS: acquired funding. VD, PA, SM-T, JS, RS, and AS: coordinated the subprojects. CD, AD, TH, TK, JP, LP, MSchä, MSchi, JS, LT, and AV: conducted the experiments. VD, PA, HC, CD, AD, TK, JP, LP, MSchä, MSchi, JS, RS, LT, AV, and AS: analyzed the data and prepared figures. VD, PA, AD, LP, MSchi, JS, RS, and AS: wrote the manuscript.

## Conflict of Interest

The authors declare that the research was conducted in the absence of any commercial or financial relationships that could be construed as a potential conflict of interest. The handling Editor declared a shared affiliation, though no other collaboration, with several of the authors (AD, SM-T).
